# Bacterial protein-oleate complexes induce ferroptosis-like cell death in colorectal cancer cells by disrupting cell membranes and inhibiting the β-catenin-GPX4 axis

**DOI:** 10.1038/s41420-026-03097-9

**Published:** 2026-04-11

**Authors:** Naeem Ullah, Abdelbasset Yabrag, Amjad Ali, Aftab Nadeem

**Affiliations:** 1 Department of Molecular Biology, Umeå University, Umeå, Sweden; 2 Umeå Centre for Microbial Research (UCMR), Umeå University, Umeå, Sweden

**Keywords:** Colon cancer, Cancer therapy

## Abstract

The tumoricidal activity of human α-lactalbumin complexes, such as HAMLET and its α-helical domain with sodium oleate, is well-documented. However, the potential of bacterial α-helical proteins to form analogous anticancer complexes remains unexplored. In the current study, we demonstrate that α-helical proteins of bacterial origin can form tumoricidal complexes with sodium oleate. Using non-hemolytic toxin A (NheA), an inactive component of the native tripartite (NheABC) toxin complex from *Bacillus thuringiensis*, we show that NheA, upon mixing with sodium oleate (NheA-O), forms potent tumoricidal complexes against colorectal cancer cells. The NheA-O complex binds to the plasma membrane of tumor cells, disrupting the function of cellular organelles and ultimately causing cell death. Mechanistically, NheA-O induces ACSL4 and suppresses GPX4 expression, which ultimately leads to the accumulation of lipid peroxidation, following suppression of β-catenin signaling. The suppression of β-catenin signaling and its target proteins ultimately leads to suppression of colorectal cancer tumorigenesis. Functionally, NheA-O inhibits tumor cell migration, spheroid formation, clonogenic potential, ATP production and induces lipid peroxidation. These findings establish that bacterial α-helical proteins, like their human counterparts, can be engineered to form tumoricidal complexes with sodium oleate. Our work highlights NheA-O as a novel candidate that causes activation of ferroptosis-like cell death in target cancer cells, leading to intracellular organelles dysfunction. Moreover, NheA-O activity synergizes with RSL3, and NheA-O mediated cell death is antagonized by Fer-1, indicating the role of NheA-O in inducing ferroptosis-like cell death. Overall, these results describe NheA-O as a novel therapeutic agent to combat tumorigenesis by targeting tumor cell membrane and proteasomal degradation of GPX4 to trigger ferroptosis-like cell death and expands the paradigm of tumoricidal protein-lipid complexes functionality across biological kingdoms.

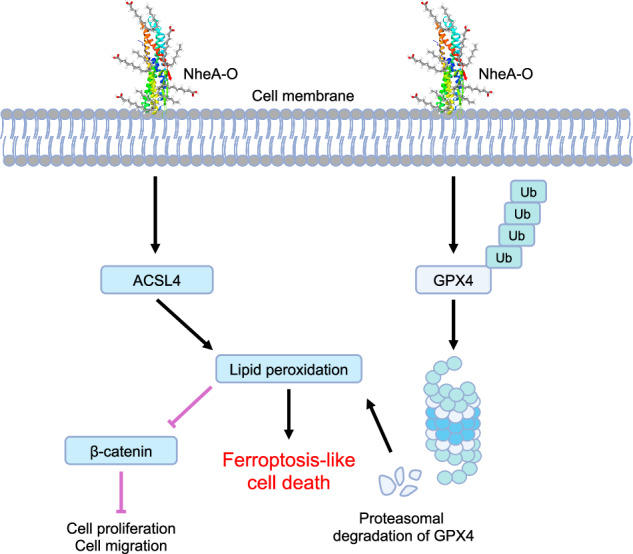

## Highlights

1. NheA mixed with sodium oleate (NheA-O) forms potent tumoricidal complexes against colorectal cancer cells.
2. NheA-O complex binds directly to the plasma membrane of tumor cells and causes dysfunction of intracellular organelles.
3. NheA-O complex suppresses tumor cell growth, cell migration, ATP production and clonogenic potential.
4. NheA-O complex triggers ferroptosis-like cell death, synergizes with RSL3 and induces lipid peroxidation.
5. NheA-O complex targets β-catenin-GPX4 axis to suppress colorectal tumorigenesis.

## Introduction

The global incidence of cancer is increasing rapidly, making it the second most common cause of mortality worldwide. There were 20 million new cancer cases and nearly 10 million cancer-related deaths in 2022, and it is expected that the number of new cases will reach more than 35 million by 2050 [1]. Colorectal cancer is the third most common cancer in the world. This disease often outsmarts existing therapies due to tumor cells’ remarkable genetic flexibility [2]. The adenomatous polyposis coli (APC) gene, mutated in hereditary colorectal tumors and over 80% of sporadic ones, impairs β-catenin degradation. This leads to β-catenin’s nuclear localization, binding to T-cell factor/lymphoid enhancer factor (TCF/LEF) transcription factors, and increased transcription of cancer-driving target genes, that subsequently activates tumor cell proliferation [3–5]. β-catenin signaling pathway has been shown to play a critical role in cancer metastasis, tumor microenvironment, angiogenesis, chemoresistance and suppression of ferroptosis. Importantly, β-catenin higher nuclear expression levels correlate with poor overall survival and disease-free survival of colorectal cancer patients [5, 6]. Depletion of β-catenin gene or inhibition of β-catenin signaling pathway with specific inhibitors has been shown to suppress colorectal cancer progression and metastasis [3, 7, 8]. Based on the critical role of β-catenin signaling pathway in colorectal cancer progression and due to its continuous activation in both early and later stages of cancer highlighting the importance of β-catenin as an attractive and potential therapeutic target. Therefore, suppressing β-catenin signaling or its downstream targets during colorectal cancer progression and metastasis represents a promising strategy for developing novel anticancer therapeutics.

Cell death is an essential process for the development and maintenance of homeostasis in organisms, and its imbalance can bring about cancer onset and malignant progression. Cell death is primarily categorized into necrosis and programmed cell death. The latter is a controlled mechanism that includes apoptosis, necroptosis, pyroptosis, autophagy, and ferroptosis [9]. Ferroptosis is recognized as iron-dependent form of cell death that is morphologically, biochemically and genetically distinct from apoptosis, autophagy and various forms of necrosis [10]. Glutathione peroxidase 4 (GPX4) has been recently shown as one of the key regulators of ferroptosis. It utilizes reduced glutathione to convert lipid hydroperoxides to lipid alcohols, which mitigate lipid peroxidation and inhibit ferroptosis [11]. GPX4 increased expression in tumors significantly correlates with tumorigenesis, metastasis, chemoresistance and poor survival of cancer patients [11, 12]. Ferroptosis can be induced by various therapeutic agents, and the suppression of ferroptosis is associated with cancer cells survival, chemotherapy resistance and cancer progression [13].

Protein-lipid complexes composed of α-lactalbumin and oleic acid display anticancer activities against different types of cancer cells [3, 7, 14]. HAMLET (human alpha-lactalbumin made lethal to tumor cells) is the first member of such complexes formed from alpha helical rich proteins and oleic acid. The molecular mechanisms involved in HAMLET-induced tumor cell death has been extensively characterized [3, 15–18]. It has been proposed that HAMLET binds to tumor cell membrane, causing tumor cell membrane perturbation, leading to activation of ion fluxes and MAPK signaling [19]. Upon internalization to the tumor cell cytoplasm, it interacts with various organelles following its rapid translocation to tumor cell nuclei, and binding to histones [20].

To date, alpha-lactalbumin and its structurally related proteins, including lysozyme, have been shown to form tumoricidal complexes upon mixing with oleic acid [21]. Moreover, it has been recently reported that synthetic alpha-helical peptides from alpha-lactalbumin and Sar1 can form tumoricidal complexes when mixed with sodium oleate [16, 22]. The therapeutic activity of such complexes has been demonstrated in a mouse model of bladder cancer and has been further verified in patients with bladder cancer [3, 22]. These findings raise the question of whether proteins from non-mammalian origin may have the ability to form tumoricidal complexes with sodium oleate, similar to HAMLET, alpha1-Oleate, or Sar1-Oleate. To address this question, we formed tumoricidal complexes of sodium oleate with NheA (non-hemolytic toxin A), an inactive member of the tripartite bacterial toxin NheABC, which is produced by both pathogenic (*B. cereus*) and non-pathogenic (*B. thuringiensis*) bacterial species within the *Bacillus cereus* group. NheA on its own lacks the ability to bind target host cells [23]. However, upon interaction with NheB and NheC, it forms an active tripartite complex that has the ability to lyse target host cells [24]. In the present study we proposed that NheA forms tumoricidal complexes upon mixing with sodium oleate. The tumoricidal complexes of NheA-O have similar biological activity to HAMLET-like protein-lipid complexes. Importantly, we discovered that the NheA-O complex induces ferroptosis-like cell death in colorectal cancer cells. Moreover, we showed that NheA-O complex-mediated ferroptosis-like cell death was increased in colorectal cancer cells in combination with ferroptosis inducer, RSL3 and partially rescued by the ferroptosis inhibitor Fer-1, suggesting a novel role for the NheA-O complex in ferroptosis-like cell death induction. Mechanistically, we report that NheA-O complexes bind to the tumor cell membrane and causes an increase in the expression of ACSL4 and a decrease in the expression of GPX4, leading to an increase in lipid peroxidation. This then lead to decrease in the expression of β-catenin and its target proteins. Importantly, the NheA-O complex caused a decrease in the activity of β-catenin, which was partially rescued by the ferroptosis inhibitor Fer-1. Functional studies reveal that NheA-O caused an increase in lipid peroxidation, and induced cell death in colorectal cancer cells. Furthermore, colorectal cancer cells treated with NheA-O showed a decrease in ATP production, cell proliferation, cell migration and spheroid formation. Overall, these results highlight the NheA-O complex as a novel therapeutic agent with the potential to trigger ferroptosis-like cell death, thereby suppressing colorectal cancer progression. Our findings shed light on the NheA-O complex as a novel inducer of ferroptosis-like cell death and point to its importance in the suppression of colorectal cancer tumorigenesis.

## Results

### NheA-O forms biologically active tumoricidal complexes

**Fig. 1:**
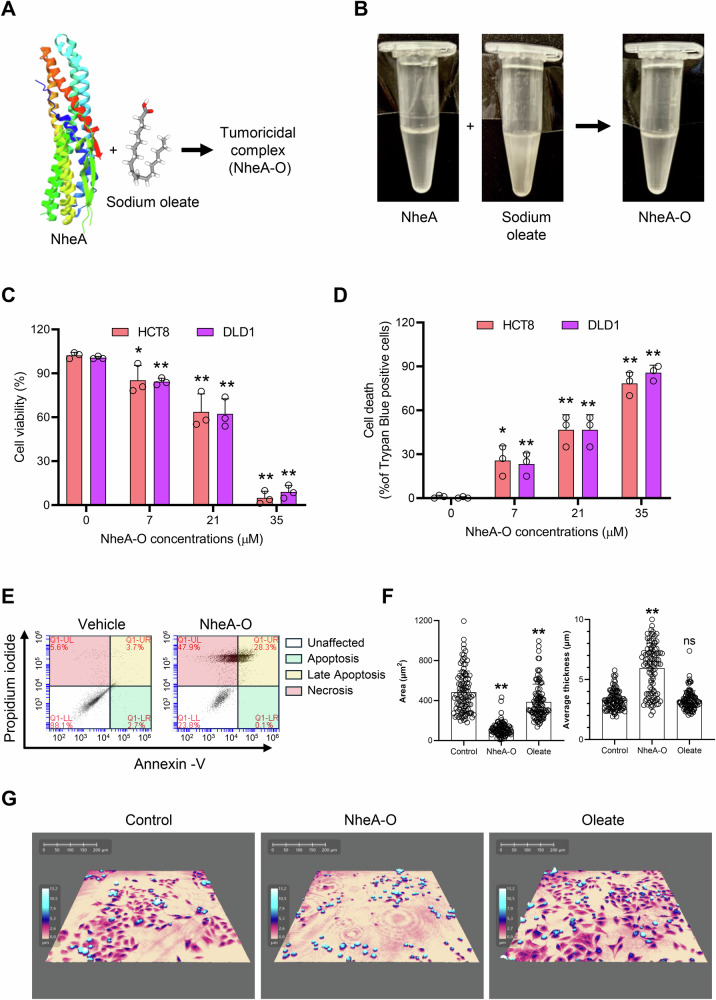
NheA forms tumoricidal complexes with oleate. **A** Schematic representation of NheA-O complex formation. The model depicts crystal structure of NheA (PDB: 4K1P) and 3D model of sodium oleate (PubChem CID = 23665730). **B** Images of NheA-O and sodium oleate solutions prepared in PBS. **C**, **D** NheA-O causes concentration-dependent cell death in HCT8 and DLD1 colorectal cancer cells (n = 3). Cell death was quantified by (**C**) Presto Blue and (**D**) Trypan Blue assays. Bar graphs show mean ± s.d. Significance was determined from three biological replicates using unpaired Student’s t-test (controls vs treated). **p < 0.01, *p ≤ 0.05. **E** Representative plot from flow cytometry analysis of two biological replicates shows induction of DLD1 cell death in response to NheA-O. **F** Histograms depict changes in cell area and optical thickness. Data points represent individual DLD1 cells (n = 50 random cells from two biological replicates); bar graphs show mean ± s.d. Significance was determined from replicates using a one-way analysis of variance (ANOVA) with Dunnett’s post-test against untreated control cells. **p < 0.01, *p ≤ 0.05. **G** Changes in cell morphology of DLD1 cells were recorded by holographic microscopy. Representative holographic microscopy images from three biological replicates of DLD1 cells with or without NheA-O. Color gradients (cream to white) indicate cell thickness, with white representing the thickest regions.

HAMLET has been previously shown to induce cell death in various cancer cells. Moreover, it has been reported that, unlike the β-sheet domain of alpha-lactalbumin, its alpha-helical domains can form potent tumoricidal complexes upon mixing with sodium oleate [16, 25]. To determine if a bacterial protein rich in alpha helices may form similar tumoricidal complexes, we purified the NheA protein of *Bacillus* cereus (Fig. S1A), which is rich in alpha helices, using an *Escherichia coli* expression system and mixed it with sodium oleate (Fig. 1A). The formation of NheA-O complex was evaluated by visual inspection using turbidity analysis (Fig. 1B) or CD analysis (Fig. S1B). The protein NheA caused reduction in the turbidity of sodium oleate solution (Fig. 1B), a characteristic feature known for the formation of tumoricidal protein-lipid complexes [26]. Moreover, the CD data shows secondary-structure alterations in the presence of sodium-oleate consistent with minor conformational changes (Fig. S1B). We then exposed colorectal carcinoma cells to increasing concentrations of NheA-O for 3 hours. NheA-O caused concentration-dependent cell death in both HCT8 and DLD1 colorectal carcinoma cells, as shown by a reduction in the metabolic activity of the tumor cells, quantified by a decrease in Presto Blue fluorescence (Fig. 1C). To determine if NheA-O attacks the plasma membrane of tumor cells, we performed a trypan blue exclusion assay and observed that NheA-O causes a concentration-dependent increase in trypan blue-positive cells (Fig. 1D). Importantly, the cytotoxic activity of NheA-O was similar to that of bovine alpha-lactalbumin-oleate complexes (BAMLET) (Fig. S1C, D). Cell death was further confirmed by flow cytometry, which revealed a significant increase in late apoptotic and necrotic cell populations compared to vehicle-treated controls (Figs. 1E and S2A). We also tested the effect of NheA-O on non-transformed colon cells CCD18-Co, which were less sensitive to NheA-O challenge at the concentration that effectively killed human colon carcinoma cells, DLD1 and HCT8 (Figs. 1C, D and S2B). By SparkCyto image analysis, we observed time-dependent changes in DLD1 cell morphology in response to NheA-O. Importantly, the individual components of the complex, NheA (35 μM) or oleate (175 μM), failed to induce changes in tumor cell morphology (Fig. S2C). To quantify the morphological changes in response to NheA-O, we performed holographic image analysis. Holographic imaging of DLD1 cells showed an increase in the optical thickness and a reduction in total cell area in response to NheA-O (Figs. 1F–G and S2D). Importantly, NheA-O caused minimal changes in the cell morphology of non-transformed colon cells CCD18-Co (Fig. S2D). Together, these results suggest that NheA-O forms tumoricidal complexes that are as potent as BAMLET.

### Disruption of intracellular organelles by NheA-O

**Fig. 2:**
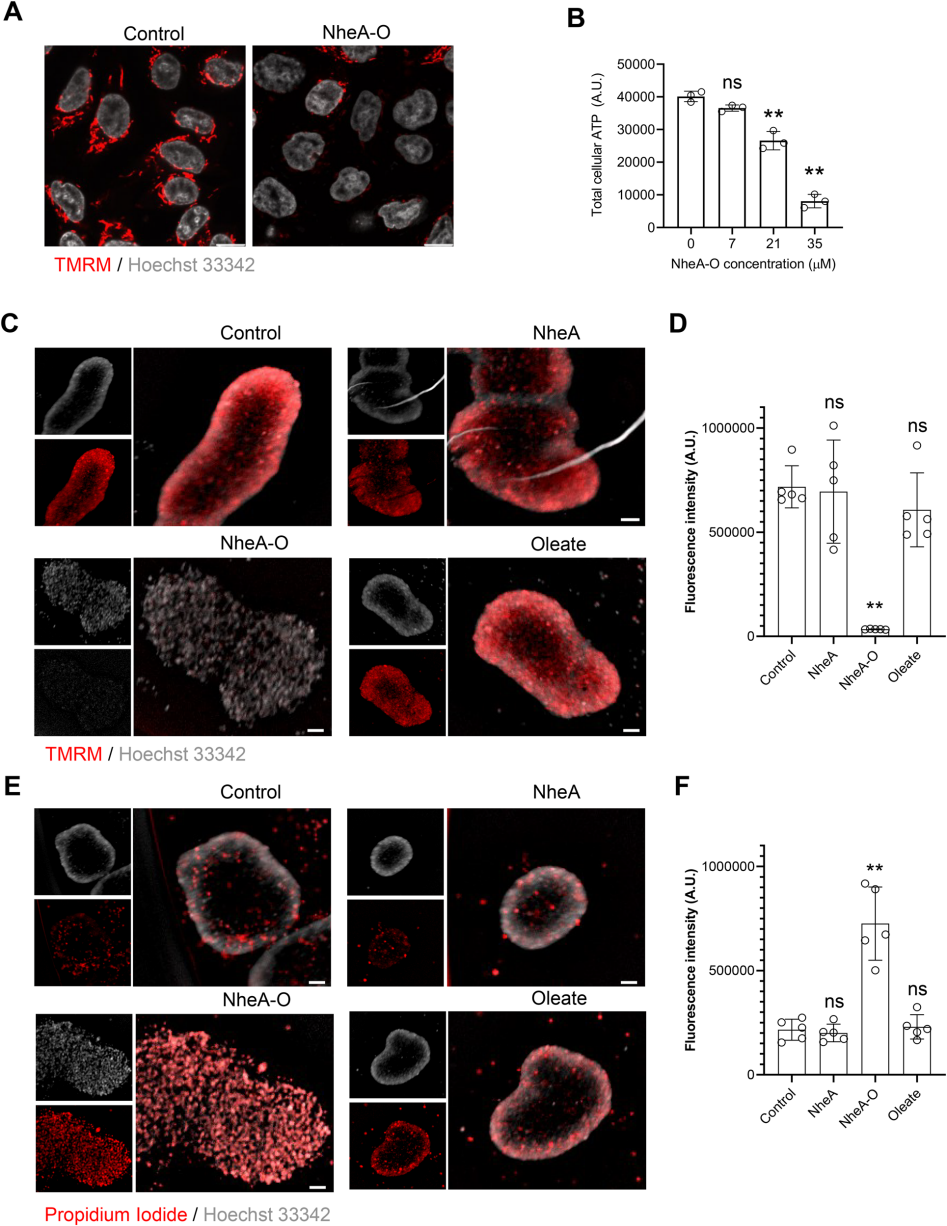
NheA-O induces metabolic paralysis in tumor cells. **A** DLD1 cell monolayers were treated with NheA-O (35 μM, 1 hour) and stained with TMRM (red). Nuclei were counterstained with Hoechst 33342 (gray). NheA-O reduced TMRM fluorescence intensity. Confocal microscopy images are representative of two biological replicates. Scale bars = 10 µm. **B** DLD1 cells were exposed to increasing concentrations of NheA-O for 3 hours, followed by total cellular ATP measurement. NheA-O caused a concentration-dependent decrease in ATP levels. Bar graphs show mean ± s.d. Significance was determined from three biological replicates using a one-way analysis of variance (ANOVA) with Sidak’s multiple comparisons test (controls vs treated). **p < 0.01, ns = non-significant. **C** DLD1 3D spheroids (formed over 72 hours) were treated with NheA-O or its individual components for 3 hours and stained with TMRM (red). Nuclei were counterstained with Hoechst 33342 (gray). SparkCyto images are representative of two biological replicates. Scale bars = 50 µm. **D** Histogram showing reduced TMRM fluorescence intensity in NheA-O-treated spheroids. Data points represent individual spheroids. Data is representative of two biological replicates. Bar graphs show mean ± s.d. Significance was determined from five individual spheroids using a one-way analysis of variance (ANOVA) with Dunnett’s multiple comparisons test (controls vs treated). **p < 0.01, ns = non-significant. (controls vs treated). **E** DLD1 3D spheroids (formed over 72 hours) were treated with NheA-O or its individual components for 24 hours and stained with propidium iodide (red). Nuclei were counterstained with Hoechst 33342 (gray). SparkCyto images are representative of two biological replicates. Scale bars = 50 µm. **F** Histogram showing increased propidium iodide fluorescence intensity in NheA-O-treated spheroids. Data points represent five individual spheroids. Data is representative of two biological replicates. Bar graphs show mean ± s.d. Significance was determined using one-way ANOVA with Dunnett’s post hoc test (controls vs treated). **p < 0.01, ns = non-significant.

To determine whether cell membrane binding and intracellular uptake of NheA-O may cause dysfunction of intracellular organelles, including lysosomes and mitochondria, we stained DLD1 cells with acridine orange or TMRM and then exposed them to NheA-O (Figs. 2A and S3A, B). NheA-O caused a significant reduction in the number of acidic vacuoles stained by acridine orange (Fig. S3B). Moreover, we observed an overall decrease in TMRM staining in NheA-O-treated DLD1 cells (Fig. 2A). To determine if mitochondrial dysfunction could lead to a reduction in total cellular ATP, we exposed DLD1 cells to increasing concentrations of NheA-O, following measurement of total cellular ATP. Consistent with the loss of mitochondrial function, NheA-O caused a concentration-dependent decrease in total cellular ATP (Fig. 2B). To determine if NheA-O would induce loss of mitochondrial potential in a complex 3D cell culture model, we prepared spheroids from DLD1 cells and exposed them to NheA-O. Similar to the DLD1 cells grown as a monolayer, NheA-O caused an overall reduction in TMRM staining in 3D spheroids formed from DLD1 cells (Fig. 2C, D). Importantly, the individual components of NheA-O failed to induce a loss of mitochondrial potential in the 3D spheroids (Fig. 2C, D). Consistent with the loss of mitochondrial function in the cells grown in 3D spheroids, we observed an increase in cell death, as quantified by an increase in propidium iodide uptake in the 3D spheroids exposed to NheA-O (Fig. 2E, F). Together, the results suggest that NheA-O causes dysfunction of intracellular organelles in the targeted tumor cells.

### NheA-O targets tumor cell membrane

**Fig. 3:**
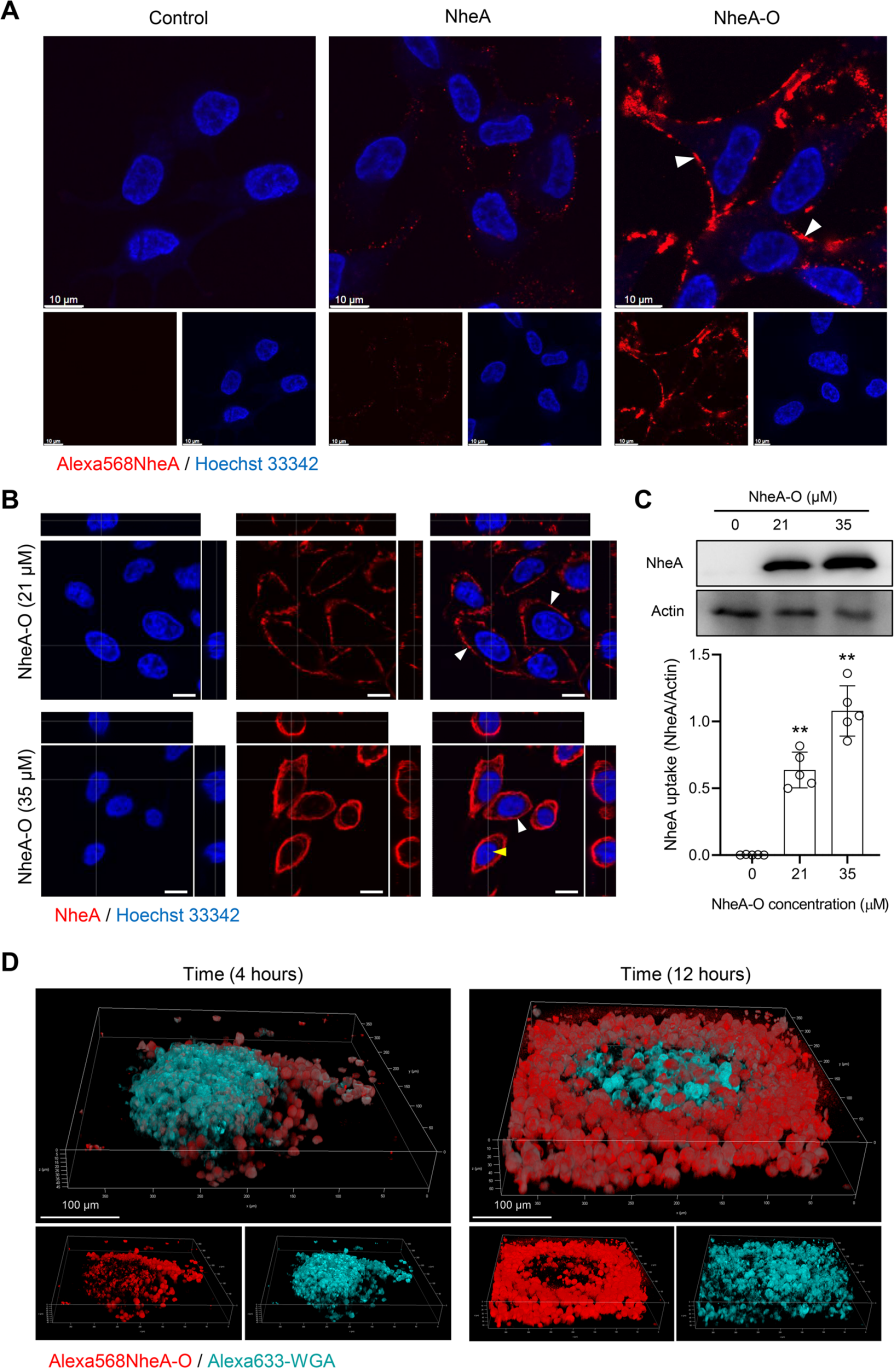
NheA-O associates with tumor cell membranes and intracellular organelles of tumor cells. **A** DLD1 colorectal cancer cells were exposed to Alexa568NheA (21 μM, red) or Alexa568NheA-O (21 μM, red) for 1 hour. Nuclei were counterstained with Hoechst 33342 (blue), and live-cell imaging was performed using a confocal microscope. White arrowheads indicate the association of NheA-O with the cell membrane. Confocal microscopy images are representative of two biological replicates. Scale bars = 10 µm. **B** DLD1 cells treated with increasing concentrations of NheA-O. NheA (red) was detected using anti-NheA monoclonal antibodies. Nuclei were counterstained with DAPI (blue). White arrowheads indicate membrane-associated NheA, while yellow arrowheads denote nuclear accumulation. Confocal microscopy images are representative of two biological replicates. Scale bars = 10 µm. **C** Western blot analysis of DLD1 cells exposed to increasing concentrations of NheA-O. Data analysis suggests concentration dependent uptake of NheA-O. Actin was used as an internal loading control. Bar graphs show mean ± s.d. Significance from biological replicates (n = 5) was determined using unpaired Student’s t-test (controls vs treated). **p < 0.01 **D** Representative images of DLD1 spheroids treated with Alexa568-NheA-O (red) at two different time-points. DLD1 cells in the spheroid were labelled with Alexa633-WGA (cyan). Confocal microscopy images are representative of two biological replicates. Scale bars = 100 µm.

To address if the direct binding of NheA-O to tumor cell membrane is responsible for inducing morphological changes in tumor cell membrane, we exposed DLD1 cells to Alexa568NheA or Alexa568NheA-O (21 μM, 1 hour). By confocal microscopy, we observed that majority of Alexa568NheA-O was associated with cell membrane of the affected cell, compared to the Alexa568NheA alone (Fig. 3A). To confirm the cell membrane association of the NheA in the treated tumor cell, we exposed DLD1 cells to increasing concentrations of NheA-O, following fixation. NheA was detected using monoclonal antibodies (Fig. 3B). Consistent with the Alexa568NheA-O treated cells, we observed that the majority of NheA-O (21 μM, 1 hour) was bound to the tumor cell membrane (Fig. 3B). By 3D reconstruction of the confocal images, in addition to NheA-O cell membrane association, we observed that small fraction of NheA-O (35 μM, 1 hour) was localized in the cytosol and nuclei of the affected tumor cells (Fig. 3B). Western blot analysis further confirmed concentration dependent increase of NheA-O uptake in treated DLD1 cells (Figs. 3C and S11). To address if NheA-O can penetrate deep into the core of 3D spheroid, we prepared 3D spheroids from DLD1 cells and exposed them to Alexa568NheA-O. Images were acquired live at two time-points (4 and 12 hours). By live cell confocal microscopy, we observed an increase in the uptake and penetration of NheA-O deep into the core of 3D spheroids (Fig. 3D).

**Fig. 4:**
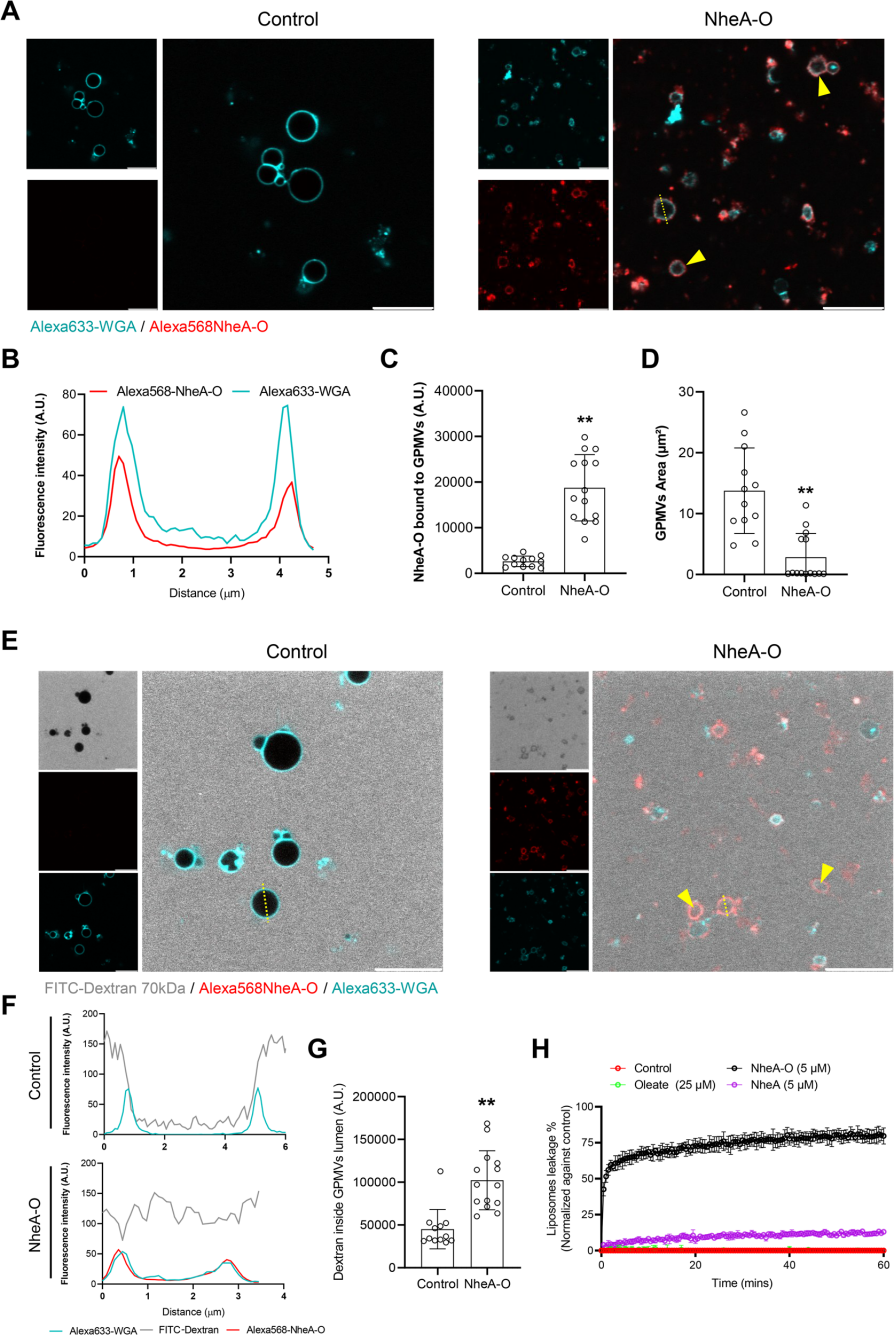
NheA-O associates with GPMVs isolated from tumor cell membranes. **A** GPMVs isolated from DLD1 cells were treated with Alexa568NheA-O (1 hour, red). Membranes were stained with Alexa633-WGA (cyan). Yellow arrowheads indicate Alexa568NheA-O association with GPMVs. Confocal microscopy images are representative of two biological replicates. Scale bars = 10 µm. **B** Line plot (yellow dotted line) in (**A**) shows NheA-O distribution across GPMV membranes. **C** Histogram quantifying Alexa568NheA-O binding to GPMV membranes. Data points represent fluorescence intensity per individual GPMVs (n = 12–14). Data is representative of two biological replicates. Significance was determined using unpaired Student’s t-test (controls vs treated). **p < 0.01 **D** Histogram showing reduced total GPMV area following NheA-O treatment. Data points represent individual GPMVs (n = 12–14). Data is representative of two biological replicates. Significance was determined using unpaired Student’s t-test (controls vs treated). **p < 0.01 **E** Yellow arrowheads highlight increased GPMV permeability induced by Alexa568NheA-O. Confocal microscopy images are representative of two biological replicates. Scale bars = 10 µm. **F** Line plot (yellow dotted line) in (**E**) illustrates the colocalization of NheA-O (red), FITC-Dextran (gray), and Alexa633-WGA (cyan) membrane staining across GPMVs. **G** Histogram demonstrating increased FITC-Dextran influx into GPMV lumens in the presence of NheA-O. Data points represent individual GPMVs (12-14). Data is representative of two biological replicates. Bar graphs show mean ± s.d. Significance was determined using unpaired Student’s t-test. **p < 0.01, *p ≤ 0.05. **H** NheA-O causes time-dependent increase in liposomes leakage prepared from DLD1 epithelial cell lipid extract. Liposome leakage was quantified by an increase in fluorescence of sulforhodamine B release from the leaked liposomes to the media upon exposure to NheA-O (5 μM). NheA (5 μM) and sodium oleate (25 μM) were used as a control. The experiment was repeated twice with similar results (n = 3; technical replicates).

To determine whether the direct binding of NheA-O to the tumor cell membrane is responsible for inducing morphological changes in the tumor cell membrane (Fig. 1F, G and S2C, D), we prepared giant plasma membrane vesicles (GPMVs) from DLD1 cells and exposed them to Alexa568NheA-O (Fig. 4A). We used GPMVs as a model system for studying the interaction of NheA-O with the cell membrane because they are cell-derived vesicles that largely retain the components of the cell membrane [27]. By confocal microscopy, we observed the accumulation of NheA-O complexes across the membrane of GPMVs (Fig. 4A–C). Moreover, we observed a strong correlation between Alexa568NheA-O and Alexa633WGA-labeled GPMVs (Fig. 4B). In addition to NheA-O binding to GPMVs, we observed a reduction in the total area of the GPMVs exposed to NheA-O compared to the control (Fig. 4D). To determine whether the reduction in the area of GPMVs in response to NheA-O may lead to cell membrane permeability, we incubated GPMVs with FITC-70kDa Dextran [28], which does not permeate into the lumen of intact GPMVs (Fig. 4E). Upon exposure to NheA-O, we observed the translocation of FITC-70kDa Dextran into the lumen of the GPMVs, suggesting that NheA-O causes permeability of the cell membrane (Fig. 4E–G). To determine whether NheA-O targets tumor cell lipid membranes, we extracted lipids from the tumor cells and performed sulforhodamine B-loaded liposome assay (Fig. 4H). NheA-O caused time-dependent increase in the leakage of liposomes, as indicated by an increase in the sulforhodamine B fluorescence compared to the control or its individual components, NheA or Oleate (Fig. 4H). Taken together, our data suggests that accumulation of NheA-O on the target tumor cell membrane causes perturbation of the tumor cell membrane, that ultimately leads to activation of cellular mechanisms involved in cell death.

### NheA-O causes suppression of β-catenin signaling in colorectal carcinoma cells

**Fig. 5:**
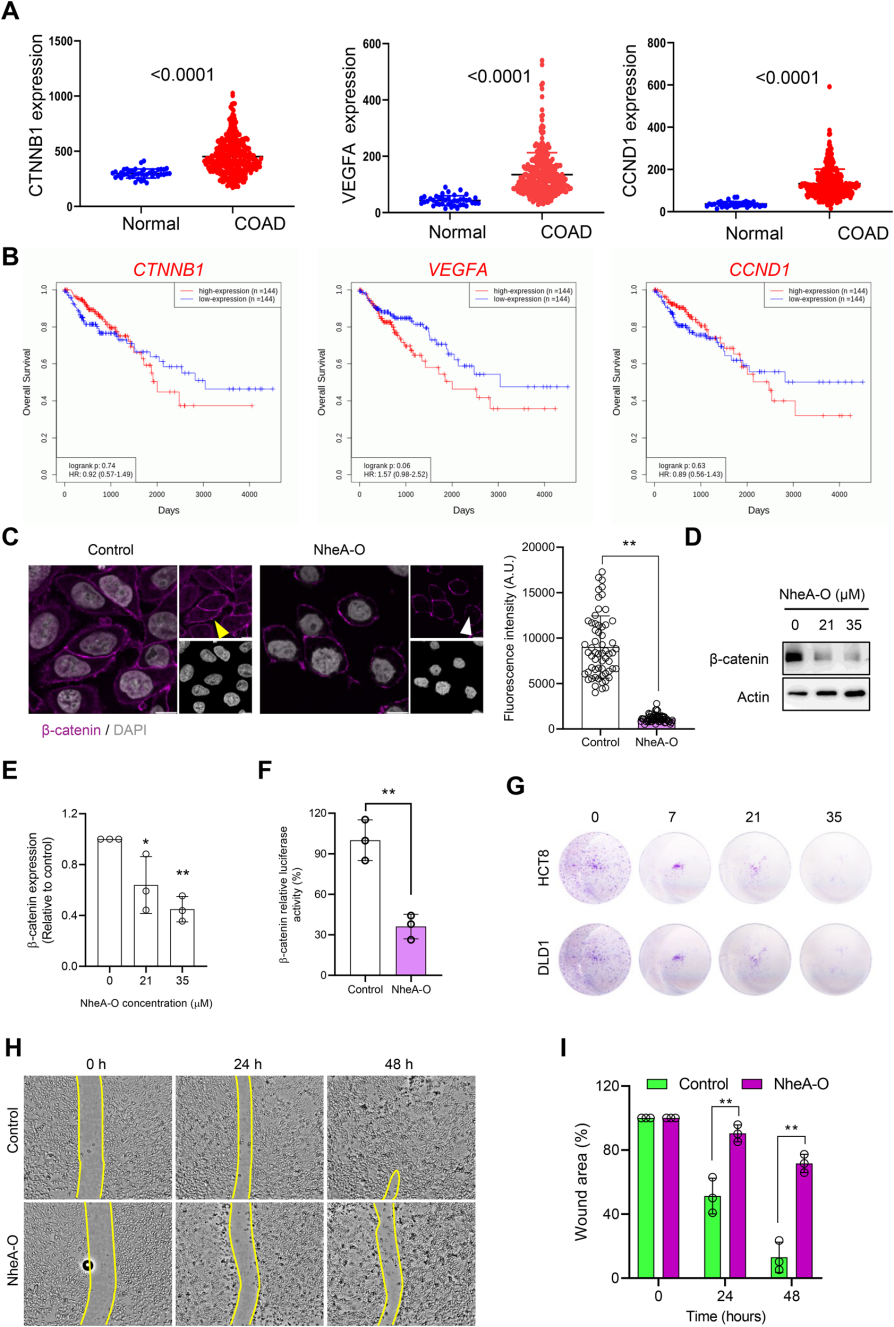
Targeting β-catenin signaling by NheA-O complexes in colorectal cancer cells. **A** TCGA analysis identified overexpression of β-catenin and its downstream signaling molecules in colorectal cancer patients. **B** Survival plots suggest that higher expression of *CTNNB1* and its downstream target genes *VEGFA* and *CCND1* correlates with poor survival in colorectal cancer patients. **C** Yellow arrowheads indicate reduced nuclear accumulation of β-catenin (magenta) in NheA-O-treated cells. Nuclei were counterstained with DAPI (gray). The histogram (right) shows reduced β-catenin fluorescence intensity in NheA-O-treated cells. Data points represent nuclear fluorescence intensity of β-catenin in DLD1 cells (n = 50 cells from two biological replicates). Significance was determined using unpaired Student’s t-test. Scale bars = 10 µm. **D**, **E** Western blot analysis of DLD1 cells exposed to increasing concentrations of NheA-O. NheA-O caused decrease in the expression of β-catenin. Actin was used as a loading control. Histogram in (**E**) indicates decrease in the expression of β-catenin in response to NheA-O. Bar graphs show mean ± s.d. Significance was determined from three biological replicates using a one-way analysis of variance (ANOVA) with Sidak’s multiple comparisons test (controls vs treated). **p < 0.01, *p < 0.05. **F** TCF/LEF (T-cell factor/lymphoid enhancer binding factor) reporter activity, quantified via TOP-flash firefly luciferase assay, shows reduced luciferase activity in HCT8 cells (n = 3; biological replicates). Significance was determined from three biological replicates using unpaired Student’s t-test. **G** NheA-O inhibited HCT8 and DLD1 cell proliferation in a concentration-dependent manner, as quantified by colony-forming assay. Data is representative of three biological replicates. **H**, **I** NheA-O inhibited DLD1 cell migration in a time-dependent manner. Images in (**H**) are representative of three biologically independent experiments. Histograms in (**I**) quantify the scratch area (n = 3 from three biological replicates). Bar graphs show mean ± s.d. Significance was determined using unpaired Student’s t-test. **p < 0.01, *p ≤ 0.05.

To determine the role of β-catenin signaling in colorectal cancer progression, we analyzed TCGA data (Fig. 5A). Our analysis revealed elevated expression of *CTNNB1* (β-catenin) and its target genes *VEGFA* (VEGF) and *CCND1* (Cyclin D1) in colorectal cancer patients. Importantly the expression of *CTNNB1, VEGFA*, and *CCND1* was positively correlated with each other (Fig. S4A). Furthermore, higher expression of these genes correlated with poor patient survival (Fig. 5B). Aberrant expression of β-catenin leads to its nuclear accumulation in APC mutant cells, thus playing an important role in tumor cell proliferation. Previously, we reported that HAMLET suppresses β-catenin signaling by activating ion fluxes in colorectal carcinoma cells [3]. To investigate whether NheA-O causes a β-catenin-dependent decrease in tumor cell proliferation, DLD1 cells were exposed to increasing concentrations of NheA-O, followed by β-catenin staining. Western blot analysis suggested a concentration dependent overall decrease in the total expression of β-catenin (Figs. 5C–E and S11). By confocal microscopy we observed a significant decrease in nuclear accumulation of β-catenin (Figs. 5C and S4B). To determine whether NheA-O inhibits β-catenin signaling in colorectal cancer cells, we exposed DLD1 cells to NheA-O and performed a TCF/LEF reporter assay to measure β-catenin activity (Fig. 5F). Importantly, the decrease in nuclear accumulation of β-catenin coincides with a decrease in its activity (Fig. 5C, F). To assess whether NheA-O downregulates β-catenin downstream targets, we treated DLD1 cells with NheA-O and analyzed the expression of two key targets: Cyclin D1 (by Western blot) and VEGF (by confocal microscopy). NheA-O treatment significantly reduced the expression of both Cyclin D1 and VEGF in DLD1 cells (Figs. S4C–F and S11). Consistent with the decrease in β-catenin activity in response to NheA-O, we observed a concentration-dependent decrease in tumor cell colony formation in both DLD1 and HCT8 cells (Figs. 5G and S5A). Moreover, we also observed a decrease in DLD1 cells migration as shown by reduction in wound healing in response to NheA-O assay (Figs. 5H, I and S5B, C). These results were then further confirmed by transwell cell migration assay (Fig. S5C). Together, the data suggest that NheA-O causes inhibition of β-catenin signaling, and tumor cell migration in colorectal cancer cells.

### NheA-O causes ferroptosis-like cell death in colorectal carcinoma cells

**Fig. 6:**
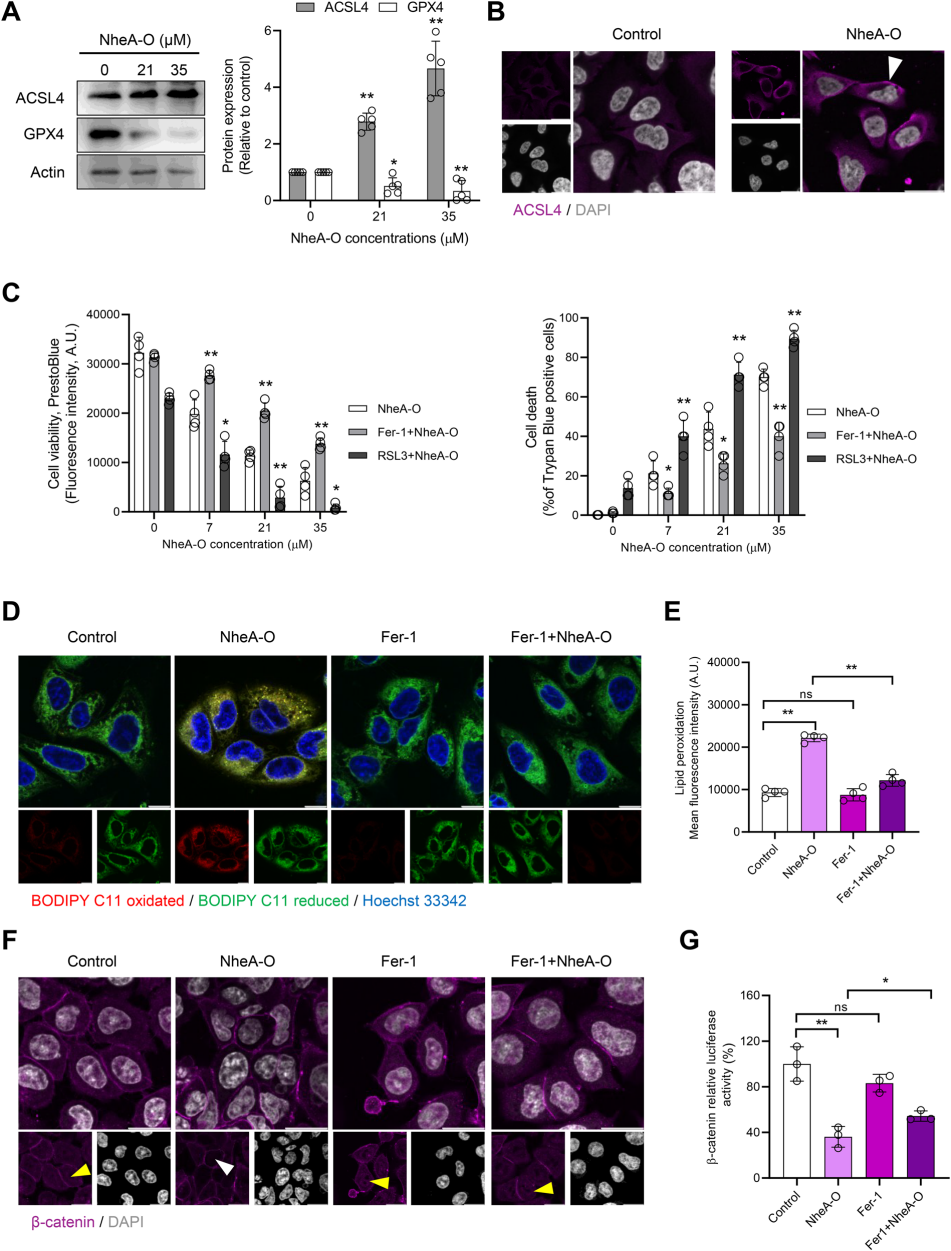
NheA-O induces ferroptosis-mediated suppression of β-catenin in colorectal cancer cells. **A** Western blot analysis of DLD1 cells exposed to increasing concentrations of NheA-O indicates concentration dependent increase in the expression of ACSL4 and decrease in the expression of GPX4. Actin was used as a loading control. Bar graph to the right shows mean ± s.d. Significance from biological replicates (n = 5) was determined using unpaired Student’s t-test (controls vs treated). **p < 0.01, *p ≤ 0.05. **B** Representative confocal microscopy images from two biological replicates indicate increase in the expression of ACSL4 in NheA-O treated cells. The arrowheads white indicates cell membrane association of ACSL4 in NheA-O treated cells. **C** NheA-O mediated cell death in DLD1 colorectal cancer cells in the presence or absence of ferroptosis inhibitor (Fer-1) or activator (RSL3). Cell death was quantified by decrease in Presto Blue staining, and an increase trypan blue-positive cells. (n = 4; biological replicates). Bar graphs show mean ± s.d. Significance was determined using unpaired Student’s t-test (NheA-O treated cells vs cells treated with corresponding concentration of NheA-O in the presence of Fer-1 or RSL3). **p < 0.01, *p ≤ 0.05. **D** Representative confocal microscopy images of NheA-O-induced lipid peroxidation (red) in DLD1 cells (n = 2 biological replicates). Ferroptosis inhibition by Fer-1 rescues NheA-O-driven lipid peroxidation. Scale bars = 10 µm. **E** Histogram showing flow cytometry quantification of lipid peroxidation in response to NheA-O in the presence or absence of Fer-1 (n = 4 biological replicates). **F** Confocal microscopy reveals that NheA-O reduces nuclear accumulation of β-catenin, a process that is rescued by Fer-1. The arrowheads yellow indicates nuclear accumulation of β-catenin, while white arrowhead indicates decrease in nuclear accumulation of β-catenin. Confocal microscopy images are representative of two biological replicates. Scale bars = 10 µm. **G** Histogram depicting TCF/LEF reporter activity (via TOP-Flash firefly luciferase assay) in HCT8 cells treated with NheA-O in the presence or absence of Fer-1 (n = 3; biological replicates). Bar graphs in (**E**, **G**) shows mean ± s.d. Significance was determined between the biological replicates using unpaired Student’s t-test. **p < 0.01, *p ≤ 0.05, ns = non-significant.

Cell death in response to various anticancer drugs is primarily categorized into necrosis and programmed cell death. Programmed cell death mainly includes apoptosis, necroptosis, pyroptosis, autophagy, and ferroptosis. Ferroptosis is recognized as an iron-dependent, non-apoptotic form of cell death driven by lipid peroxidation [10, 29]. To address if NheA-O induces apoptosis or necrosis in tumor cells, DLD1 cells were pre-incubated with the pan-caspase inhibitor, z-VAD or necroptosis inhibitor, Necrostatin-1 (Nec-1) that specifically inhibits RIPK1 [30], following cells exposure to NheA-O for three hours. Both inhibitors failed to rescue NheA-O mediated cell death, suggesting that these pathways do not play an important role in NheA-O mediated cell death (Fig. S6A, B). To investigate whether NheA-O induces ferroptosis-like cell death in tumor cells, we treated DLD1 cells with increasing concentrations of NheA-O. Western blot analysis suggested an increase in the expression of ACSL4 and a decrease in GPX4 expression (Figs. 6A and S11). The increase in the expression of ACSL4 was further confirmed by confocal microscopy (Figs. 6B and S7). Importantly, we observed an increase in the accumulation of ACSL4 on the tumor cell membrane in response to NheA-O (Fig. 6B). To investigate whether ferroptosis plays an important role in NheA-O-mediated cell death, we exposed DLD1 cells to increasing concentrations of NheA-O in the presence of the ferroptosis inhibitor ferrostatin-1 (Fer-1) or the ferroptosis activator RAS-selective lethal 3 (RSL3). Data analysis from the trypan blue, PrestoBlue, and MTS assays indicated that Fer-1 significantly inhibits NheA-O-mediated cell death, whereas RSL3 enhances it (Figs. 6C and S8A, B). In addition to preventing cell death, Fer-1 also reversed NheA-O-induced changes in cell morphology, as observed by holographic imaging (Fig. S9A, B). To determine whether NheA-O-mediated induction of ferroptosis-like cell death leads to increased lipid peroxidation, we exposed DLD1 cells to NheA-O and visualized lipid peroxidation using BODIPY C11 using confocal microscopy. Data analysis revealed an increase in lipid peroxidation in response to NheA-O, which was partially rescued by Fer-1 (Fig. 6D). These results were further confirmed by flow cytometry analysis (Fig. 6E). To investigate the role of ferroptosis in regulating β-catenin signaling, we exposed DLD1 cells to NheA-O in the presence or absence of Fer-1, following confocal microscopy. Data analysis suggested a decrease in nuclear accumulation of β-catenin, that was reversed by Fer-1 pre-treatment (Fig. 6F). Moreover, data analysis of the TCF/LEF reporter assay indicated a decrease in β-catenin activity in response to NheA-O that was partially rescued by Fer-1 (Fig. 6G). Together, these results suggest that NheA-O activates ferroptosis-like cell death, which may lead to the suppression of β-catenin signaling.

### NheA-O is associated with reduced GPX4 levels in a proteasome-dependent manner

**Fig. 7:**
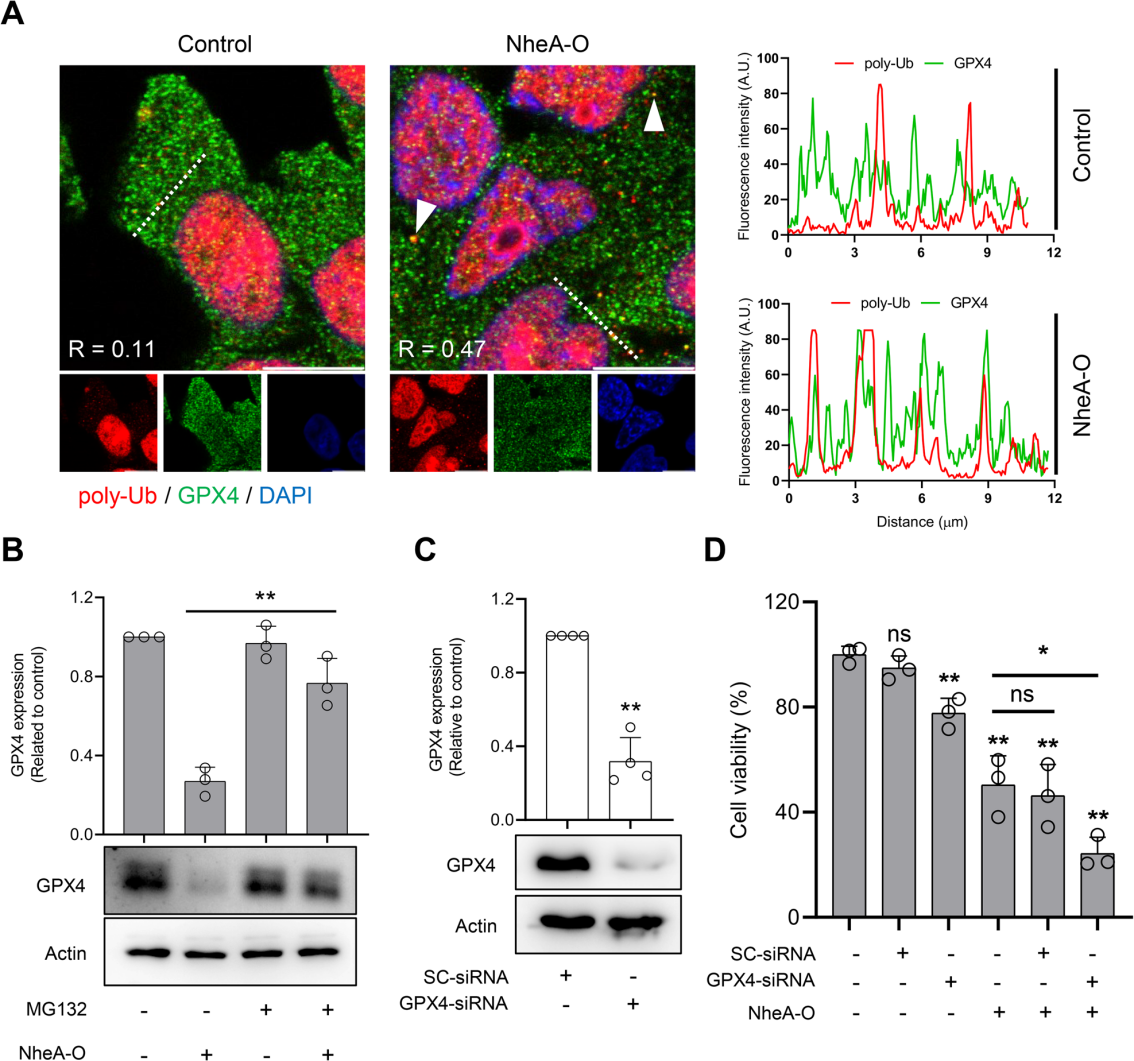
NheA-O promotes proteasomal degradation of GPX4. **A** Representative confocal microscopy images from two biological replicates indicate increase in co-localization of GPX4 (green) with poly-Ubiquitin (red) in the NheA-O treated DLD1 cells, compared to the control. White arrowheads indicate co-localization of GPX4 and poly-Ubiquitin. The line plots to the right indicate fluorescence intensity profiles of the corresponding image along the dotted white line, used for calculation of Pearson correlation co-efficient (R). **B** Western blot analysis of DLD1 cells exposed to NheA-O (21 μM) in the presence or absence of proteasome inhibitor, MG132 (20 μM) for three hours. GPX4 was detected using GPX4-specific primary antibodies. Actin was used as a loading control. Bar graph indicates quantification of Western blots from three biologically independent experiments. Data is represented as Mean ± s.d. Significance was determined using unpaired Student’s t-test. **p < 0.01, *p ≤ 0.05. **C** Western blot analysis of DLD1 cells exposed to scrambled (SC) or GPX4 siRNA for 48 hours. GPX4 was detected using GPX4-specific primary antibodies. Actin was used as a loading control. Bar graph indicates quantification Western blots from four biologically independent experiments. Data is represented as Mean ± s.d. Significance was determined using unpaired Student’s t-test. **p < 0.01. **D** Depletion of GPX4 with siRNA for 48 hours, had an additive effect on the reduction in DLD1 cell viability in response to NheA-O. Cell viability was quantified by MTS cell viability assay. Bar graphs show mean ± s.d. from three biologically independent experiments. Significance was determined using unpaired Student’s t-test (controls vs treated or NheA-O vs SC-siRNA and GPX4-siRNA). **p < 0.01, *p ≤ 0.05.

The ubiquitin-proteasome system (UPS) represents a primary protein degradation pathway in eukaryotes [31]. Recent studies have shown that some ferroptosis inducers can increase the ubiquitination of GPX4 [32]. To investigate whether the decrease in GPX4 expression in response to NheA-O may involve the UPS, we exposed DLD1 cells to NheA-O and assessed the co-localization of GPX4 with poly-Ubiquitin via confocal microscopy (Fig. 7A). Data analysis suggested an increase in the co-localization of GPX4 with poly-Ubiquitin in response to NheA-O, raising the possibility of UPS-mediated degradation of GPX4. To further test the role of proteasome in the degradation of GPX4, we used the proteasome inhibitor, MG132. Western blot analysis showed that the decrease in GPX4 expression in response to NheA-O was partially rescued by MG132 (Fig. 7B). To determine whether depletion of GPX4 contributes to induction of ferroptosis in response to NheA-O, we depleted GPX4 using GPX4-specific siRNA. Western blot analysis suggested a decrease in GPX4 expression in cells exposed to GPX4 siRNA compared to DLD1 cells treated with scrambled siRNA (Figs. 7C and S11). Furthermore, the importance of GPX4 in NheA-O-mediated cell death was investigated using an MTS cell viability assay. DLD1 cells treated with scrambled siRNA or GPX4 siRNA (48 hours) were exposed to NheA-O for three hours, followed by the cell viability assay (Fig. 7D). Data analysis suggested a decrease in cell viability in response to GPX4 siRNA compared to scrambled siRNA. Moreover, GPX4 siRNA-treated DLD1 cells showed additive cell death with NheA-O, suggesting that GPX4 depletion sensitizes DLD1 cells to NheA-O treatment (Fig. 7D). Taken together, our results suggest that tumor cell exposure to NheA-O is associated with reduced GPX4 levels in a manner that involves the proteasome, and this may contribute to induction of ferroptosis-like cell death.

## Discussion

In this study, we introduce a novel therapeutic approach by demonstrating that bacterial α-helical proteins, specifically non-hemolytic toxin A (NheA) from the biotechnologically important bacterium *B. thuringiensis*, can form potent tumoricidal complexes with sodium oleate (NheA-O) against colorectal cancer cells. While the tumoricidal activity of human α-lactalbumin complexes, such as HAMLET, is well-established, the potential of bacterial α-helical proteins to form analogous anticancer complexes remains unexplored. Our findings reveal that NheA-O disrupts colorectal cancer function by targeting tumor cell membrane. NheA-O mediated disruption of tumor cell membrane leads to activation of ACSL4 and proteasomal degradation of GPX4 leading to an increase in lipid peroxidation, triggering induction of ferroptosis-like cell death. This process then leads to a decrease in the expression of β-catenin and its target proteins. We observed strong binding of NheA-O to tumor cell membranes compared to NheA alone, consistent with a previously published report [23]. This binding was further confirmed in giant plasma membrane vesicles (GPMVs) derived from colorectal cancer cells, where NheA-O accumulated unevenly, suggesting localization to specific membrane compartments. GPMVs capture the compositional complexity of cancer cell plasma membranes and are free from intracellular organelle contamination [33]. A liposome leakage assay indicated that NheA-O, similar to HAMLET, targets lipid membranes [17]. However, unlike HAMLET and alpha1-O, which primarily accumulates in tumor cell nuclei [34], NheA-O remains bound to the cell membrane, making it the primary site of action. This membrane accumulation enables NheA-O to activate ferroptosis in targeted cancer cells.

Ferroptosis is a recently described cell death mechanism driven by iron-dependent peroxidation of polyunsaturated phospholipids in cell membranes [35]. It is triggered when GPX4 function is impaired, leading to lethal lipid peroxide accumulation [36]. Ferroptosis plays a critical role in non-apoptotic cancer cell death, resensitizing tumors to chemotherapy and supporting tumor suppressor gene function, making it a vital mechanism against cancer metastasis, invasion, and chemoresistance [37]. Our results suggest that NheA-O induces a ferroptosis-like cell death in colorectal cancer cells, as supported by observations of increased lipid peroxidation, synergy with the ferroptosis inducer RSL3, and partial rescue by the ferroptosis inhibitor ferrostatin-1 (Fer-1). Our findings are consistent with reports that certain ferroptosis inducers can promote GPX4 degradation via the proteasome [32], although direct evidence for ubiquitination of GPX4 in response to NheA-O, such as ubiquitin pull-down assays or degradation kinetics, remains to be established. We propose a model in which NheA-O-induced membrane perturbation correlates with reduced GPX4 levels, potentially contributing to ferroptosis-like cell death. However, the detailed molecular mechanisms, including any role for ubiquitination, require further mechanistic investigation.

Colorectal cancer cells treated with NheA-O exhibited decreased GPX4 expression, indicating that NheA-O disrupts the GPX4-dependent pathway. GPX4 is a critical oncogene associated with tumor progression and reduced patient survival; its inhibition is linked to ferroptosis induction and tumor suppression [37]. We propose that NheA-O causes membrane perturbation, which may lead to the ubiquitination of GPX4 and its subsequent proteasomal degradation, a finding consistent with recent report that a proteasomal inhibitor can rescue GPX4 degradation in response to ferroptosis inducers [32]. Importantly, the proposed sequence linking membrane perturbation, β-catenin modulation, GPX4 reduction, and ferroptosis-like death remains associative and requires further investigation. Beyond inducing cell death, NheA-O reduced invasiveness and tumor cell migration. Since cancer cell migration is a critical step in metastasis [38], NheA-O’s ability to block migration highlights its potential to prevent metastasis initiation. Furthermore, NheA-O suppresses the clonogenic potential of colorectal cancer cells, which is associated with tumor growth and chemotherapy resistance. By inhibiting clonogenicity, NheA-O may resensitize tumors to standard chemotherapeutic agents.

Although this study relies on in vitro 2D and 3D cell culture models to establish the mechanistic foundation of NheA-O’s tumoricidal activity, its translational potential is evident through parallels with established protein-lipid complexes like HAMLET, which has progressed from in vitro discoveries to demonstrated efficacy in animal models of colon cancer and Phase I/II clinical trials for bladder cancer, achieving tumor cell death with negligible toxicity to healthy tissues [3, 16, 20, 22]. In vitro platforms, as utilized here, are indispensable for initial molecular mechanism elucidation, high-throughput screening, and personalized therapy development. Future studies incorporating murine models will be essential to evaluate the in vivo efficacy of NheA-O, potentially accelerating this NheA-O-based anticancer therapeutic toward clinical applications as a novel, bacteria-derived agent for treating ferroptosis-resistant colorectal cancers.

In summary, our findings establish NheA-O as a novel inducer of ferroptosis-like cell death and a potential therapeutic agent for suppressing colorectal cancer progression. NheA-O targets the tumor cell membrane, leading to mitochondrial dysfunction and ferroptosis-like cell death. Mechanistically, it disrupts the β-catenin-GPX4 axis, while functionally, it suppresses tumor cell growth, ATP production, clonogenic potential, migration, and spheroid formation, and induces lipid peroxidation. This work not only establishes NheA-O as a promising therapeutic candidate but also expands the paradigm of tumoricidal protein-lipid complexes across biological kingdoms, opening new avenues for cancer therapy development.

## Materials and methods

### Chemicals

DMSO (Dimethyl sulfoxide), Formaldehyde, Triton X-100, Tween-20, Sodium dodecyl sulphate (SDS), Sodium deoxycholate and Fluoromount were from Sigma (St. Louis, MO). EDTA (ethylenediaminetetraacetic acid) and Tris (hydroxymethyl) aminomethane were from VWR (Volumetric solutions, BDH Prolabo) and DRAQ-5 was obtained from eBioscience (San Diego, CA). RPMI-1640 was from HYclone (HYCLSH30027); sodium pyruvate (11481318), non-essential amino acids (11401378), fetal calf serum (10309433), were from Thermofisher Scientific. Sodium Oleate (O7501), RSL3 (SML2234), a-Lactalbumin (L5385), Fer-1 (SML0583), Nec-1 (480065), and Sulforhodamine B (S1307) were from Sigma Aldrich. ZVAD-FMK (ALX-260-020) from Enzo Life Sciences. Hoechst 33342 (62249), DAPI (62248), BODIPY 581/591 C11 (D3861), Acridine Orange (A1301), TMRM (I34361) and Alexa Flour 568 (A10238) were from Thermofisher Scientific. FlexiTube siRNA (20 nmol, #1027418 and #1022076) from Qiagen, TransIT-X2® System (#MIR 6004, 0.75 mL) from Mirus Bio. MG132 (#474790) was from Sigma.

### Antibodies

β-actin (#A5441, WB = 1:1000, IF = 1:100) from Sigma, β-catenin (#610153, WB = 1:1000, IF = 1:100) from BD Biosciences and cyclinD1 (#MA139546, WB = 1:1000) from Invitrogen, GPX4 (#52455, WB = 1:1000, IF = 1:100) from Cell Signaling Technology and ACSL4 (66617-1-Ig, WB = 1:1000, IF = 1:100) antibodies were from Proteintech, VEGF (#sc-7269, IF = 1:100) from Santa Cruz Biotechnology, Ubiquitin (#Ab7780, IF = 1:100) from Abcam, NheA (Mab 1A8, WB = 1: 5000; IF = 1:100), HRP conjugated goat-anti-rabbit (#AS09 602, 1:5000 dilution) was from Agrisera and rabbit-anti-mouse (#K5007, 1:5000) was from Dako. For immunofluorescence, secondary antibodies conjugated with Alexa Fluor 488 donkey anti-mouse (#A21202, 1:200) and Alexa Fluor 555 goat anti-mouse (#A21422, 1:200) were from Thermofisher scientific.

### Cloning and purification of NheA protein

The *nheA* gene (UniProt code: Q81EZ8) was amplified by PCR amplification using genomic DNA extracted from *B. cereus* strain, ATCC 14579. The resulting PCR product was digested with (BspHI +EcoRl) and was subsequently inserted into pET His-TEV. The engineered construct was expressed in Rosetta (DE3) cultured in LB broth supplemented with 50 μg/mL kanamycin and 10 μg/mL tetracycline. To induce expression, 0.4 mM isopropyl 1-thio-β-d-galactopyranoside (IPTG) was introduced when the OD600nm reached 0.6, and the culture was allowed to grow over night at 18 °C. Bacteria were harvested by centrifugation at 4° C and the NheA protein was purified on Ni-NTA resin, followed by cleavage of the tagged N-terminal 6-histidine using TEV protease and re-purified through the Ni-NTA resin (Thermo Scientific) to remove the His-tag protein. The purity of the protein was investigated with Coomassie staining using SDS-PAGE (Fig. S1A).

### Production of NheA-oleate complexes

For the production of NheA-oleate complexes, purified NheA was mixed with sodium oleate in phosphate buffer saline (NaCl, 6.8 g /L, Na_2_HPO_4_x2H_2_0, 4.8 g/L; and KH_2_PO_4_, 1.3 g/L, pH7.2). The solution was vortexed at room temperature and immediately used for cellular experiments. The stoichiometry of the NheA protein to sodium oleate was kept to 1:5 ratio as recently described [39].

### Circular dichroism spectroscopy

Far-UV circular dichroism (CD) spectra of NheA protein and its respective NheA-Oleate complexes were recorded using a Jasco J-720 spectropolarimeter (Jasco, Japan) at 25 °C. Protein samples (3 µM) and sodium oleate (15 µM) were incubated overnight at 25 °C in phosphate buffer (pH 7.4). Measurements were conducted in a 0.1 cm path length quartz cuvette with a bandwidth of 2 nm. Spectra were collected over the 190–260 nm range, averaged across five scans, and corrected by subtracting the corresponding buffer baseline. All measurements were performed under identical conditions to ensure comparability across variants. The reproducibility of spectra was confirmed by repeated scans, and no significant baseline drift was observed.

### Cellular assays

Colorectal carcinoma cells (DLD1, and HCT8) and non-transformed CCD18-Co cells were purchased from American Type Culture Collection (ATCC) and cultured in RPMI-1640 with non-essential amino acids (1:100), 1 mM sodium pyruvate, 50 μg/mL Gentamicin and 5–10% fetal calf serum (FCS) at 37 °C, 5% CO_2_. For cell death experiment, cells were grown on 96-well plate (1 × 10^4^/well, Tecan Group Ltd) overnight. Cells were incubated with NheA-O complexes in serum-free RPMI-1640 at 37 °C. FCS was added after 1 hour. Cell toxicity was quantified 3 hours after the NheA-O treatment by two biochemical methods: Cell viability was quantified by Presto Blue fluorescence (Invitrogen, A13262), or MTS (3-(4,5-dimethylthiazol-2-yl)-5-(3-carboxymethoxyphenyl)-2-(4-sulfophenyl)-2H-tetrazolium) assay (Promega, G3582) and cell permeability was quantified by using Trypan Blue exclusion assay. Cellular ATP content was measured with an ATPLite kit (PerkinElmer, #6016943). ATP estimation is widely used as a biochemical method for cell viability, based on the assumptions that living cells produce ATP and it is indispensable for cellular life [40]. Presto Blue fluorescence or MTS absorbance was measured using a microplate reader (Spark, Tecan). The cell death in the presence of inhibitors, Fer-1 (1.25, 2.5, 5, 10, 20, and 40 μM), RSL3 (0.621, 1.25, 2.5, 5, 10, and 20 μM), Nec-1 or zVAD (1.25, 2.5, 5, 10, and 20 μM) was performed by preincubating the DLD1 cells with the inhibitor for 30 minutes following their exposure to NheA-O.

### Holographic microscopy

Holographic microscopy was performed using the HoloMonitor® M4 (Phase Holographic Imaging AB) equipped with a motorized stage to investigate the changes in colorectal carcinoma cells or non-transformed CCD18-Co cells exposed to NheA-O complexes. DLD1, and CCD18-Co cells (1×10^4^/well) were grown overnight in 96-well plates using RPMI-1640 medium. The following day, DLD1 cells were first pre-treated with Fer-1 (10 μM) for 30 minutes at 37 °C with 5% CO₂. The cells were then exposed to either NheA, sodium oleate or NheA-O complexes for 60 minutes at 37 °C with 5% CO₂ following image acquisition using the HoloMonitor® M4. This system generates label-free images reconstructed into three-dimensional holograms. Quantitative measurements, such as average cell thickness and area, were extracted using Hstudio™ software. Data was analyzed using GraphPad Prism software.

### Preparation of giant plasma membrane vesicles

GPMVs were produced from human colon cancer cells using a chemical vesiculation method [27]. Briefly, DLD1 and HCT cells were seeded in a 25 cm² flask and allowed to reach 70–80% confluency. The cells were washed five times with PBS to remove residual media, followed by two additional washes with GPMV buffer (10 mM HEPES [2.38 g/L], 150 mM NaCl [8.75 g/L], and 2 mM CaCl₂ [0.22 g/L], pH 7.4) to ensure proper preparation. A freshly prepared GPMV induction solution containing 25 mM PFA and 2 mM DTT in GPMV buffer was added to the cells and incubated for 2–3 hours at 37°C with 5% CO₂ to induce GPMV formation. The GPMV-rich cellular supernatants were transferred to a 15 mL Falcon tube and centrifuged at 100 rcf for 10 minutes to remove larger debris. The supernatant was collected and centrifuged again at 20,000 rcf for 1 hour at 4°C. In the next step, the supernatant was aspirated, and the pellet was resuspended in 1 mL PBS, followed by another centrifugation at 20,000 rcf for 1 hour at 37 °C. After aspiration of the supernatant, the GPMV-containing pellets were resuspended in PBS. To investigate NheA-O binding to GPMVs, we labelled GPMV membranes with Alexa Fluor™ 633-WGA (1 µg/mL) by incubating for 30 minutes at 4°C in the dark. To assess GPMV permeability, we used FITC-dextran (1 µg/mL). Labelled GPMVs (with either Alexa Fluor™ 633-WGA or FITC-dextran) were then exposed to Alexa568-NheA-O for 60 minutes, followed by imaging with a confocal microscope. Images were acquired on a Leica SP8 inverted confocal system (Leica Microsystems) equipped with an HC PL APO 63×/1.40 oil immersion lens.

### Extraction of epithelial cell lipids for liposome leakage assays

Lipids were extracted by the Folch method from 5 × 75 cm^2^ confluent flasks of DLD1 cells as recently described [41]. Briefly, DLD1 cells were grown to 90–100% confluency. Cells were washed with PBS, trypsinized, and neutralized with fresh complete RPMI medium, followed by centrifugation at 350 rcf for 10 minutes. The supernatant was discarded, and the pellet was resuspended in 3 mL of chloroform/methanol (1:2) at room temperature by vertexing every 5 minutes for 30 minutes to solubilize lipids. Subsequently, 1 mL of chloroform and 2 mL of H₂O were added, vortexed, and centrifuged at 1300 rcf for 10 minutes at room temperature. The bottom organic phase was carefully transferred to a new glass tube. This extraction step was repeated three to five times to enhance lipid purity. The final organic phase was transferred to 5 mL glass tubes and solvents were evaporated under a nitrogen flux (0.5–1 psi) and the dried lipid film was weighed ( ~ 5 mg) and stored at −20 °C until use. To prepare calcein-, or sulforhodamine B encapsulated liposomes for leakage experiments, 70 mg of calcein was added to deionized H_2_O (865 μL) and mixed with 2 N NaOH (135 μL) solution in a 1.5 ml microcentrifuge tube, or 50 mM sulforhodamine B was added to deionized H_2_O. The dried lipid film (5 mg) was hydrated either in calcein or sulforhodamine B solution. The lipid suspension was extruded through polycarbonate membranes (0.1 μm) using the Avanti Mini-Extruder (Avanti Polar Lipids, Alabaster, AL). The unbounded calcein or sulforhodamine B was removed from the loaded liposomes using a Sephadex G-50 column [42]. For the liposomes leakage assay sulforhodamine B loaded liposomes were diluted 4 times in PBS following their exposure to NheA-O (5 μM) or its individual components, NheA (5 μM), or sodium oleate (35 μM). The kinetics of liposomes leakage was investigated using Spark microplate reader at an excitation and emission wavelengths 485 nm and 530 nm for calcein, and an excitation 540 and emission at 620 for sulforhodamine B.

### Confocal microscopy

For live cell confocal microscopy, DLD1 cells were grown on a coverslip bottomed, 18-well chamber slides (2 × 10^4^/well, ibidi) and treated with Alexa568NheA or Alexa568NheA-O for 1 hour at 37°C, 5% CO_2_. The unbound protein and protein-lipid complexes were washed with serum free media, following staining of the nuclei with Hoechst 33342, following image acquisition.

For lipid peroxidation experiment, DLD1 cells were preincubated with ferroptosis inhibitor, Fer-1 for 30 minutes following incubation with NheA-O for 1 hour. Cells were stained with BODIPY 581/591 C11 (Thermo Scientific) for 30 min. The nuclei were counterstained with Hoechst 33342, following image acquisition.

Lysosomal permeability and mitochondria depolarization was studied by seeding DLD1 cells in 18-well chamber slide (2 × 10^4^/well, ibidi). The next day, cells were treated with NheA-O for 60 minutes following staining with acridine orange (1 mg/mL) or TMRM (1 μM) for 30 min. Nuclei were counterstained with Hoechst 33342 (1 μM), following image acquisition with SP8 confocal microscope.

All the samples were examined using a ×63/1.4 plan Apo λs lens or Leica SP8 inverted confocal system (Leica Microsystems) equipped with a HC PL APO ×63/1.40 oil immersion lens. Images were captured and processed using the LasX (Leica Microsystems) and ImageJ software. The images were quantified using Image J.

For fixed cells DLD1 cells were grown on an 18-well chamber slide (2 × 10^4^/well, ibidi) overnight, followed by treatment with NheA-O in the presence or absence of Fer-1 inhibitor (10 μM). Cells were fixed with 4% paraformaldehyde (20 min), permeabilized with Triton X (0.25%, 15 min), and incubated with anti-β-catenin (1:300), anti-VEGF (1:200), anti- NheA (1:200), or anti-ACSL4 (1:200) primary antibodies overnight at 4°C, followed by incubation with corresponding Alexa-488- or Alexa-555-conjugated secondary antibodies (1:200) for 1 hour at room temperature (RT). Nuclei were counterstained with DAPI (5 min, RT). Cells were imaged on a Leica SP8 inverted confocal system (Leica Microsystems) equipped with an HC PL APO ×63/1.40 oil immersion lens.

### Preparation and treatment of 3D spheroids

DLD1 cells (5×10³ cells/well) were seeded into 96-well flat-bottom plates (Greiner Bio-One) in Cancer Stem Premium Medium (ProMab) for 72 hours to allow complete spheroid formation. The spheroids were exposed to NheA-O or its individual components for 24 hours. To assess cytotoxicity and mitochondrial depolarization, spheroids were stained with propidium iodide (6 µg/mL) and TMRM (100 nM) for 30 minutes at 37°C and imaged using a SparkCyto plate reader. Nuclei were counterstained with Hoechst 33342.

To assess NheA-O uptake in a 3D cell culture model, spheroids were prepared from DLD1 cells for 72 hours and exposed to NheA-O for 4 and 12 hours. Z-stack images were acquired with confocal microscopy, which captures multiple focal planes through the spheroid, was used to evaluate cellular distribution of NheA in spheroid.

### Flow cytometry analysis

DLD1 cells were grown in 24-well plate (5×10^4^/well), overnight at 37°C with 5% CO₂. The following day, cells were treated with NheA-O in the presence or absence of Fer1 in serum-free RPMI medium. Fer1 was added 30 minutes prior to the addition of NheA-O. DLD1 cells were incubated for 3 hours at 37°C with 5% CO₂, with 5% serum added after 1 hour of treatment. Then the cells were stained with BODIPY 581/591 C11 (5 μM) for 30 min, followed by 30 minutes incubation at 37°C with 5% CO₂. To remove excess dye, the cells were washed with PBS, following their trypsinization. Cells were analyzed using a BD Accuri™ C6 Plus Flow Cytometer. Data is presented as mean fluorescence intensity.

For cell death analysis DLD1 cells were treated with NheA-O for 3 hours. After completion of the treatment, cells were subjected to flow cytometry analysis for apoptotic or necrotic cell death measurement using Annexin V/PI (BD Pharmingen) staining according to the manufacturer’s instructions. The percentage of apoptotic cells was determined by a BD Accuri™ C6 Plus Flow Cytometer (BD Bioscience). The gating strategy used for the analysis of flow cytometry data is shown in Fig. S10.

### TCF/LEF, TOPFlash reporter assay

HCT8 colorectal carcinoma cells were transfected using polyethylenimine (PEI) with a β-catenin reporter plasmid, M50 Super 8x TOPFlash that contains TCF/LEF binding sites cloned into pTA-Luc (Clontech). The plasmid, M51 Super 8x FOPFlash, was used as a control that contained mutant TCF/LEF binding sites cloned into pGL3 (Promega). M50 Super 8x TOPFlash and M51 Super 8x FOPFlash were gifts from Randall Moon (Addgene). Cells were treated with NheA-O in the presence or absence of Fer-1 for 3 hours, 24 hours post-transfection. Luciferase activity was determined with the firefly luciferase reporter assay system (Promega) using a spark microplate reader (Tecan).

### Wound healing assay

DLD1 cells were grown in 96 well plate (3×10^3^ cells/well), overnight at 37°C with 5% CO₂. The next day scratch was introduced with pipette tip. The detached cells were extensively washed with PBS, following image acquisition with sparkCyto imaging system. Cells were treated with NheA-O for 1 hour in serum free media, following addition of serum to the wells. Images were acquired at various time points, and analyzed with Image J.

### Transwell migration assay

Transwell migration assay was performed as described previously [43]. DLD1 cells were pre-treated with vehicle and NheA-O (21 and 35 µM) for 1 hour in serum free RPMI medium. A total of 5 × 10^4^ suspended DLD1 cells treated with vehicle and NheA-O were added to upper chambers using serum free media. While lower chambers were filled with 500 μL of RPMI medium supplemented with 20% FBS and 1% Pen/Strep. After 24 hours, cells were fixed with 4% PFA for 30 minutes, followed by washing with PBS. Non-migrated cells were removed by scraping with a cotton swab, followed by another PBS wash. The migrated cells were then stained with Hoechst 33342 for 10 minutes and washed again with PBS. Migrated cells were imaged using confocal microscopy, and cell numbers were quantified using ImageJ software.

### GPX4 silencing

For GPX4 silencing, FlexiTube siRNA (#1027418) was purchased from Qiagen and experiments were performed following the manufacturer’s guidelines. Briefly, the lyophilized siRNA was resuspended in 500 µL of dH₂O to prepare a 20 nM siRNA stock. siRNA-GPX4 with a final concentration of 5 nM were mixed with 15 µL of Mirus TransIT-X2® System (#MIR 6004) in serum-free media. Negative control siRNA (#1022076, Qiagen) was used as a scrambled control. The mixture was incubated at room temperature for 5–10 minutes and then added to the wells. The following day, the old media was replaced with fresh RPMI medium supplemented with 10% FBS and 1% Pen/Strep. Forty-eight hours post-transfection, cell lysates were collected for Western blot analysis. For cell death analysis, 48 hours post-transfection cells were exposed to NheA-O for 3 hours, following MTS cell viability assay.

### Western blot analysis

DLD1 cells (5×10^5^/well) were grown overnight in 6-well plates in complete RPMI medium. The following day, the cells were treated with increasing concentrations of NheA-O at 37°C with 5% CO₂ for 3 hours. The treated DLD1 cells were washed extensively with PBS (3 times) and lysed in ice-cold lysis buffer containing 20 mM Tris-HCl pH 8, 300 mM KCl, 10% Glycerol, 0.25% Nonidet P-40, 0.5 mM EDTA, 0.5 mM EGTA, 1 mM PMSF, 1× complete protease inhibitor (Roche) and phosSTOP (Roche). Protein content of each sample was quantified using BCA analysis, according to manufacturer instructions. The cell lysates were then mixed with 4x sample buffer, boiled for 10 minutes. Based on the protein content measured with BCA assay, equal amount of protein from each sample was loaded and separated by SDS-PAGE. Proteins were then transferred to a nitrocellulose membrane. The membranes were blocked in PBST (PBS supplemented with 0.1% Tween 20) containing 5% skim milk at room temperature (RT) for 1 hour. Subsequently, the membranes were incubated overnight at 4°C with the primary antibodies, anti-NheA (1:5000 dilution), anti-actin (1:5000 dilution), anti-ACSL4 (1:3000 dilution), anti-GPX4 (1:2000 dilution), or anti-β-catenin (1:10,000 dilution). After washing with PBST, the membranes were incubated with appropriate HRP-conjugated secondary antibodies (Agrisera, #AS09602, 1:5000 dilution) in blocking buffer (5% skim milk, RT, 1 hour). Protein detection was performed using a chemiluminescence reagent (Bio-Rad), and images were acquired using the ImageQuant LAS 4000 instrument. The band intensity for each protein corresponding to its molecular weight was quantified with ImageJ.

### TCGA data analysis

For the cancer genome atlas (TCGA) analysis of colon cancer and normal colon data for *CTNNB1*, *VEGF* and *CCND1* was downloaded from OncoDB database [44]. Analysis was performed to compare *CTNNB1*, *VEGF* and *CCND1* expressions in colon cancer tissues against normal colon tissues. Survival analysis for *CTNNB1*, *VEGF* and *CCND1* was performed in colon cancer patient’s samples based on high and low expressions of *CTNNB1*, *VEGF* and *CCND1* using OncoDB database [44]. Co-expression analysis of *CTNNB1* vs *VEGFA*, *CTNNB1* vs *CCND1* and *VEGFA* vs *CCND1* was performed in colon cancer patient samples using cBioPortal for cancer genomics database [45].

### Statistical analysis

Data are shown as mean ± s.d. or s.e.m. Statistical significance was determined by one-way ANOVA, or Student’s t-test as indicated in the corresponding figure legends. The number of biological repeats is indicated in the corresponding figure legends. Statistical analysis was performed using GraphPad Prism software. Statistical significance is indicated as *p* ≤ 0.01 (**) or *p* ≤ 0.05 (*). Pearson correlation coefficient, R, was performed for co-localization analysis.

## Supplementary information

Supplementary Information

Uncropped Western Blots

## Data Availability

All data supporting the findings of this study are included in the article and its Supplementary files. Additional data are available from the corresponding author upon request.
