# Supplementary material for: Bacterial protein-oleate complexes induce ferroptosis-like cell death in colorectal cancer cells by disrupting cell membranes and inhibiting the β-catenin-GPX4 axis
Source: Cell Death Discov. 2026 Apr 11;12:182. doi: 10.1038/s41420-026-03097-9 (PMC13076691; doi:10.1038/s41420-026-03097-9)
Supplement: Supplementary file 1 — Supplementary Information [file 41420_2026_3097_MOESM1_ESM.docx]

**Supplementary Information**

**Bacterial protein-oleate complexes induce ferroptosis-like cell death in colorectal cancer cells by disrupting cell membranes and inhibiting the β-catenin-GPX4 axis**

Naeem Ullah^1,2^, Abdelbasset Yabrag^1,2^, Amjad Ali^1^, Aftab Nadeem^1,2^

^1^Department of Molecular Biology, Umeå University, SE-90187 Umeå, Sweden

^2^Umeå Centre for Microbial Research (UCMR), Umeå University, SE-90187 Umeå, Sweden

*Corresponding author:

Aftab Nadeem, e-mail: aftab.nadeem@umu.se


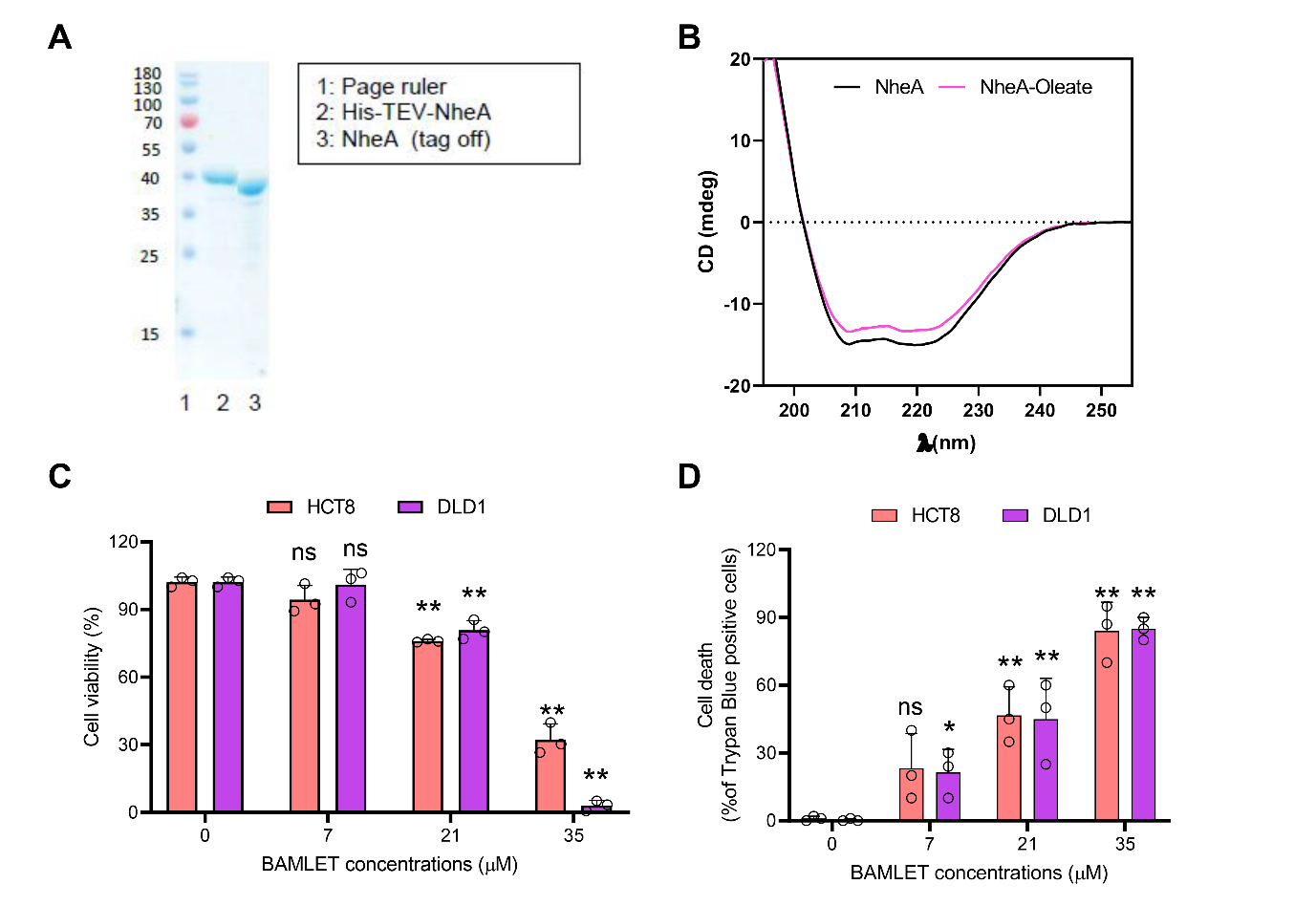


**Fig. S1: NheA forms tumoricidal complexes with sodium oleate, and BAMLET causes concentration dependent cell death in colorectal cancer cells. (A)** SDS-PAGE analysis of purified NheA. **(B)** Far-UV circular dichroism (CD) spectra of NheA protein and its respective NheA-Oleate complexes. CD spectra were obtained in phosphate buffer. The absorption intensity measured from buffer-only controls was subtracted to correct background absorption. **(C-D)** BAMLET causes concentration dependent cell death in HCT8 and DLD1 colorectal cancer cells (n = 3 from biological replicates). Cell death was quantified by (B) Presto Blue and (C) Trypan Blue assays. Bar graphs show mean ± s.d. Significance was determined from three biological replicates using unpaired Student’s t-test (controls vs treated). ***p < 0.01, *p ≤ 0.05, ns = non-significant*

**
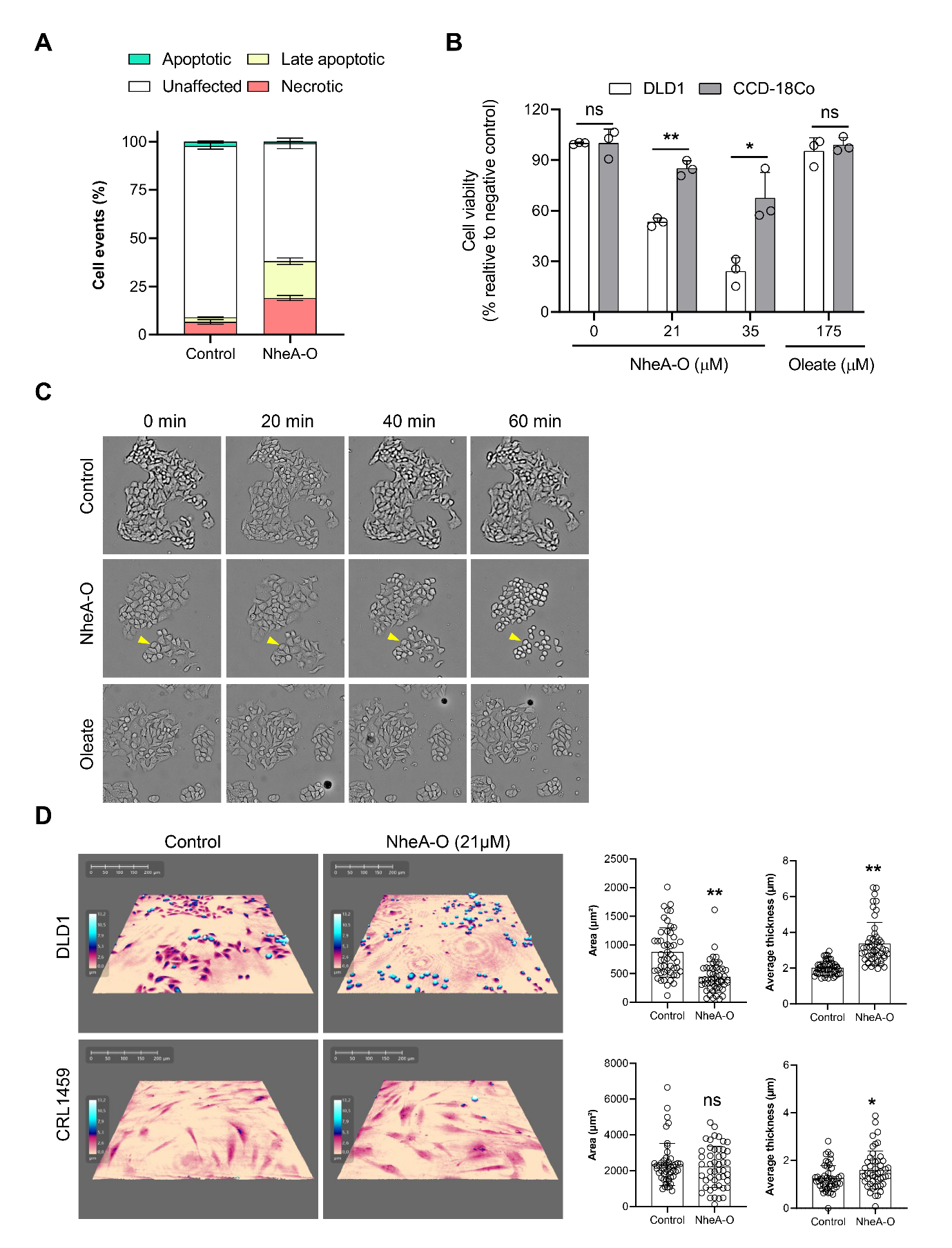
**

**Fig. S2: Non-transformed colon cells exhibit intermediate resistance to NheA-O mediated cell death. (A)** Quantification of flow cytometric data from Figure 1E. Data is expressed as mean ± s.d. of three biological experiments. **(B)** NheA-O causes concentration dependent decrease in cell viability in DLD1 colorectal cancer cells, while non-transformed colon cells are intermediately resistant to its effect (n = 3 from biological replicates). Cell viability was quantified MTS assay. Bar graphs show mean ± s.d. Significance was determined from three biological replicates using unpaired Student’s t-test. ***p < 0.01, *p ≤ 0.05, ns = non-significant* **(C)** DLD1 cells were exposed to NheA-O and images were acquired by SparkCyto imaging system. Kinetics for changes in tumor cell morphology is indicated by yellow arrowheads (n = three technical replicates). **(D)** Changes in cell morphology of DLD1 cells, and non-transformed CCD-18Co were recorded by holographic microscopy. Representative holographic microscopy images of DLD1 cells, and CCD-18Co with or without NheA-O from two biological replicates is shown. Color gradients (cream to white) indicate cell thickness, with white representing the thickest regions. Histograms to the right indicate quantification of area and thickness of control vs treated DLD1 and CCD-18Co cells. Bar graphs shows data from two biological replicates (n = 50 cells). Data is presented as mean ± s.d. Significance was determined using unpaired Student’s t-test. ***p < 0.01, *p ≤ 0.05, ns = non-significant.*

**
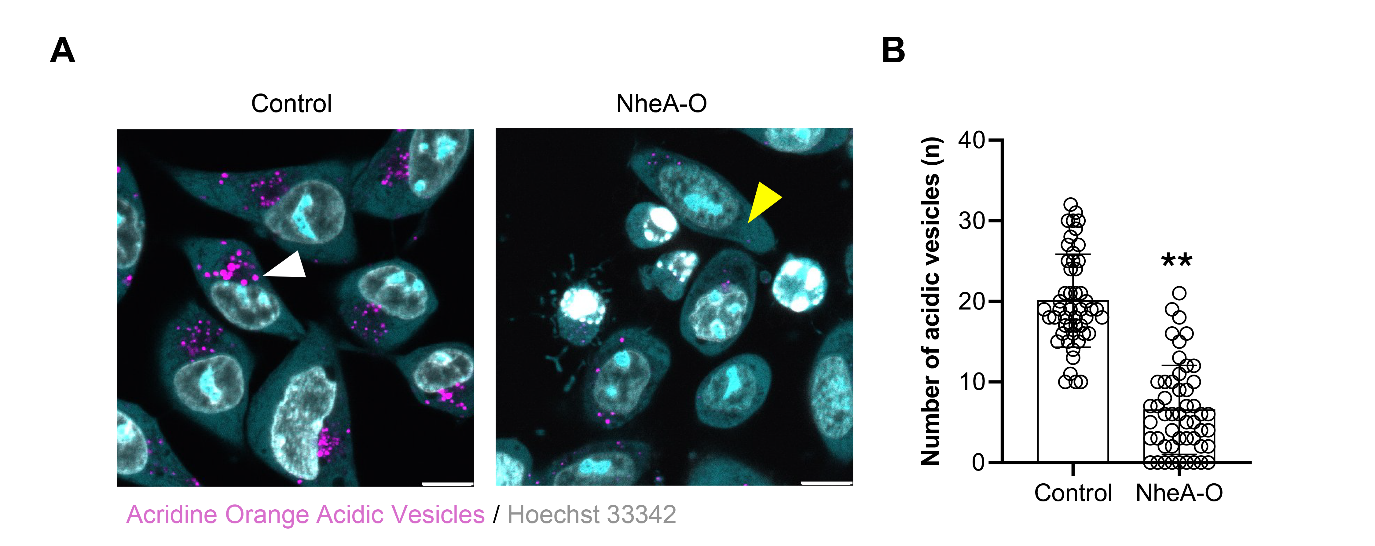
**

**Fig. S3: NheA-O causes lysosomal permeability. (A)** Representative images of confocal microscopy show reduction in number of acidic vacuoles (Arrowhead yellow) in response to NheA-O- Scale bars = 10 µm. **(B)** Bar graphs show mean ± s.d. from two biologically independent experiments (n = 50 cells). Significance was determined using unpaired Student’s t-test (controls vs treated). ***p < 0.01.*

**
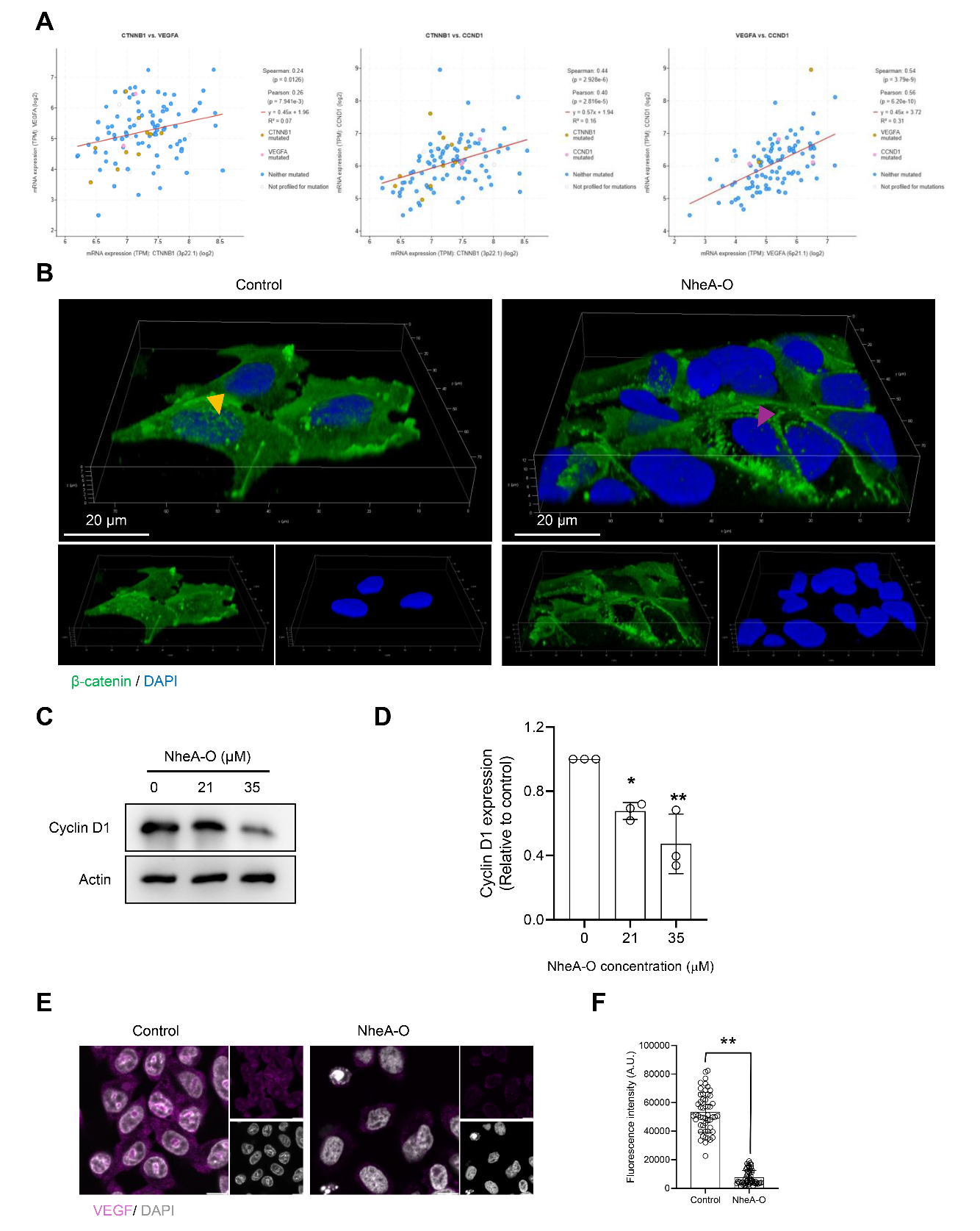
**

**Fig. S4: NheA-O causes decrease in tumor cell migration. (A)** TCGA analysis identified positive correlation between *CTNNB1* vs *VEGFA, CTNNB1* vs *CCND1*, and *VEGFA* vs *CCND1*. **(B)** Representative Z-stack images of β-catenin staining in DLD1 cells exposed to NheA-O. Yellow arrowhead indicates nuclear accumulation of β-catenin, while purple arrowhead indicates loss of nuclear accumulation of β-catenin in response to NheA-O. Images are representative of two biologically independent experiments. **(C-D)** Western blot analysis of DLD1 cells treated with NheA-O. Bar graph indicates concentration dependent decrease in the expression of Cyclin D1 in response to NheA-O. Data points represent data from three biological replicates; bar graphs show mean ± s.d. Significance was determined from replicates using a one-way analysis of variance (ANOVA) with Sidak’s post-test against untreated control cells. ***p* < 0.01, **p* ≤ 0.05. **(E-F)** Confocal microscopy data suggests that NheA-O causes reduction in the expression of VEGF. Scale bars = 20 µm. Bar graph in (F) indicates reduction in the expression of VEGF in response to NheA-O (n = 50 cells from two biological replicates). Significance was determined using unpaired Student t-test. ***p < 0.01.*


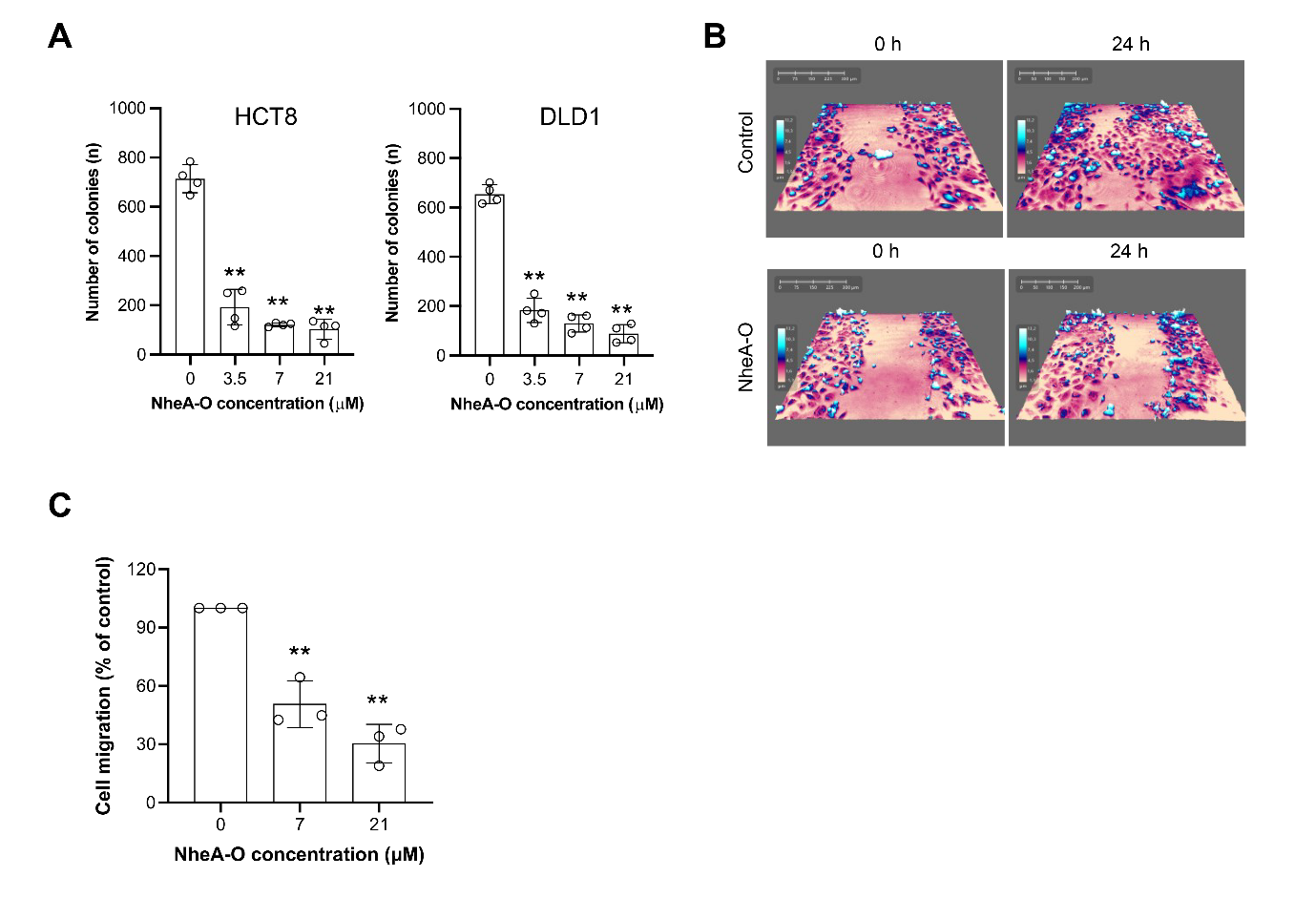


**Fig. S5: NheA-O causes decrease in tumor cell colony formation and migration. (A)** Bar graphs show a dose dependent reduction in the colony formation of HCT8 and DLD1 colon cancer cells (n = 4 from four biological replicates). Data is shown as mean ± s.d. Significance was determined using one-way ANOVA with Dunnett’s post hoc test (controls vs treated). ***p < 0.01, *p ≤ 0.05.* **B-C)** NheA-O inhibits (B) wound healing and (C) cell migration of tumor cells. Data from Holographic imaging system shown in panel (B) indicates that NheA-O inhibits wound healing in the DLD1 cells. The Bar graph indicated in panel C shows mean ± s.d. Significance was determined from three biological replicates using a one-way analysis of variance (ANOVA) with Sidak’s multiple comparisons test (controls vs treated). ***p < 0.01.*

**
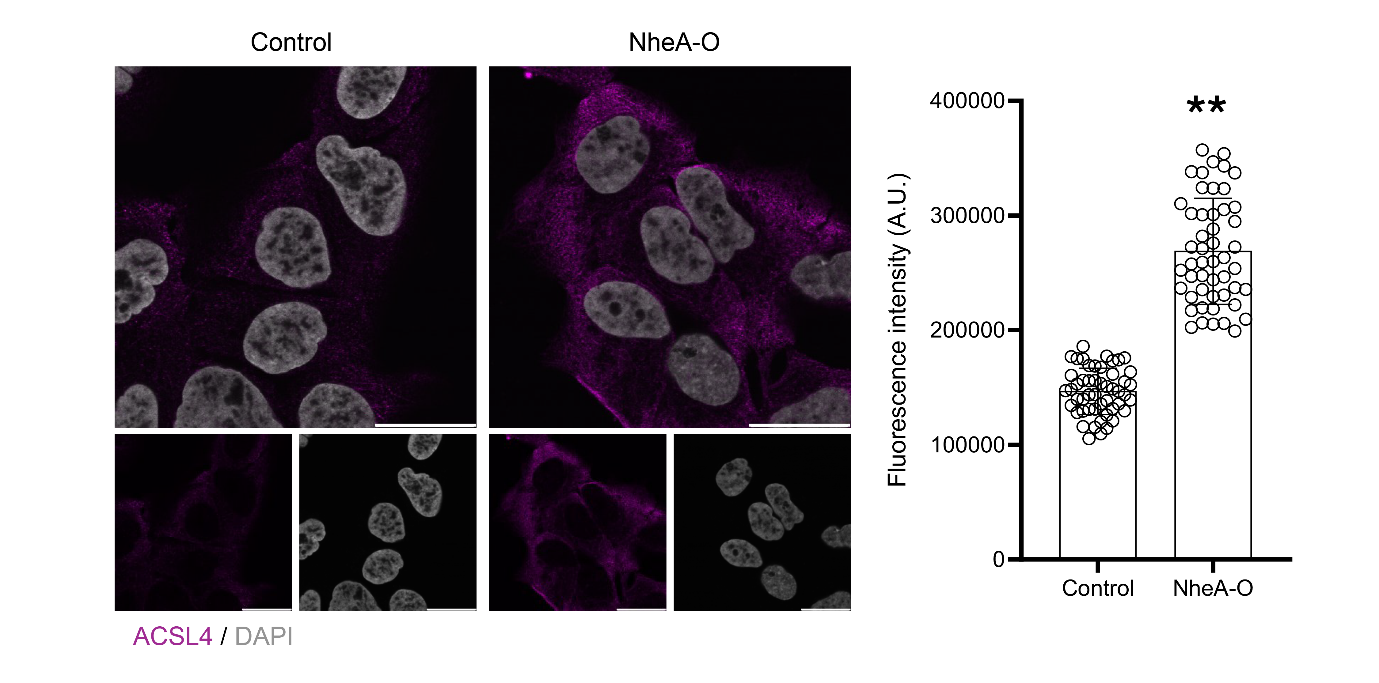
**

**Fig. S6: NheA-O causes an increase in the expression of ACSL4.**

Representative confocal microscopy images from two biological replicates indicate increase in the expression of ACSL4 in NheA-O treated cells. The histogram to the right indicates quantification of ACSL4 staining intensity (n = 50 cells from two biological replicates). Bar graphs show mean ± s.d. Significance was determined using unpaired Student’s t-test. ***p < 0.01.*

**
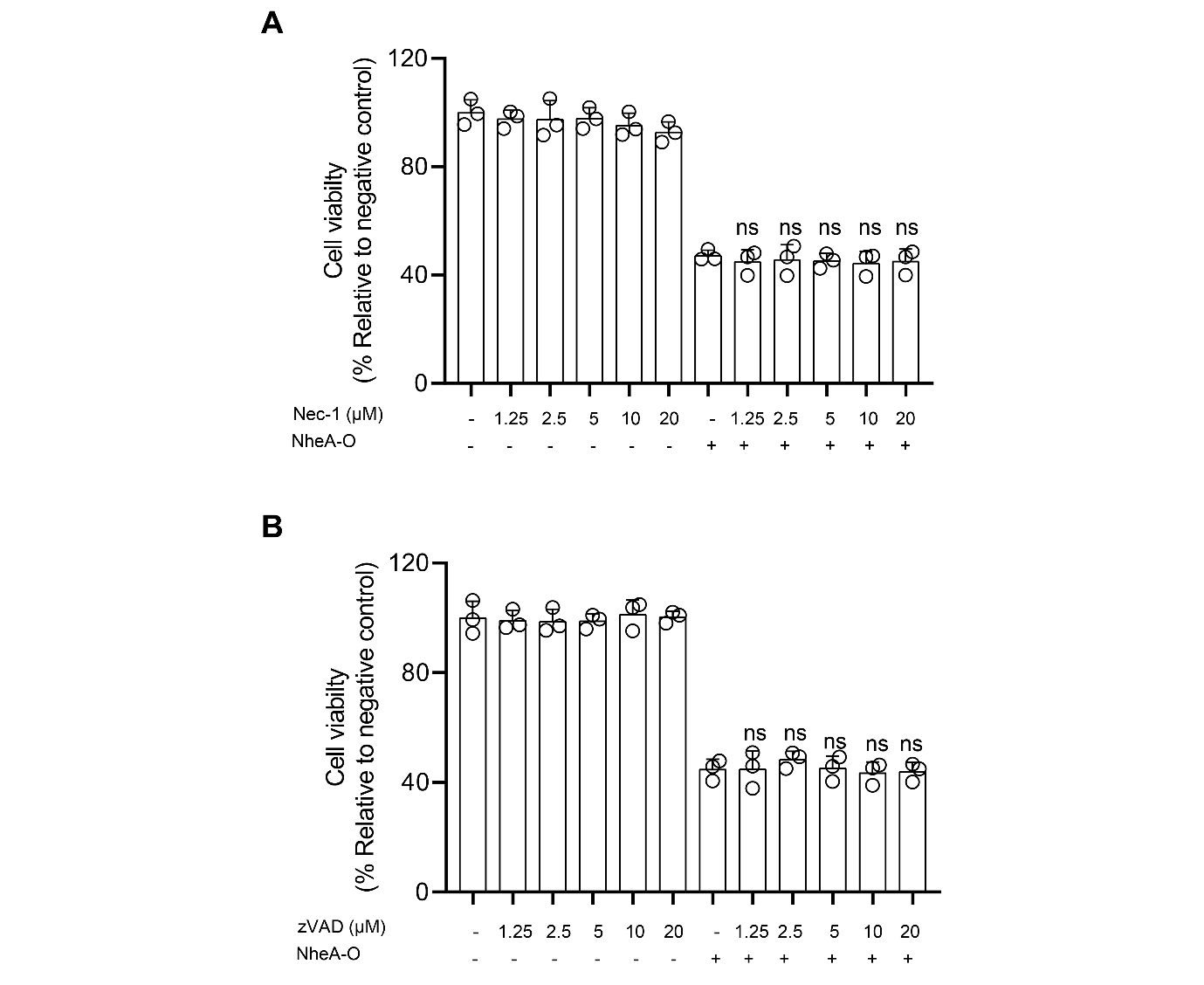
**

**Fig. S7: Tumor cell death in response to NheA-O is independent of apoptosis and necroptosis. (A-B)** NheA-O mediated cell death in DLD1 colorectal cancer cells in the presence or absence of necroptosis inhibitor (Nec-1) or pan-caspase inhibitor (zVAD). Cell viability was quantified by MTS cell viability assay (n = 3 biological replicates). Bar graphs show mean ± s.d. Significance was determined from replicates using a one-way analysis of variance (ANOVA) with Dunnett’s post-test against NheA-O treated cells. *ns = non-significant.*


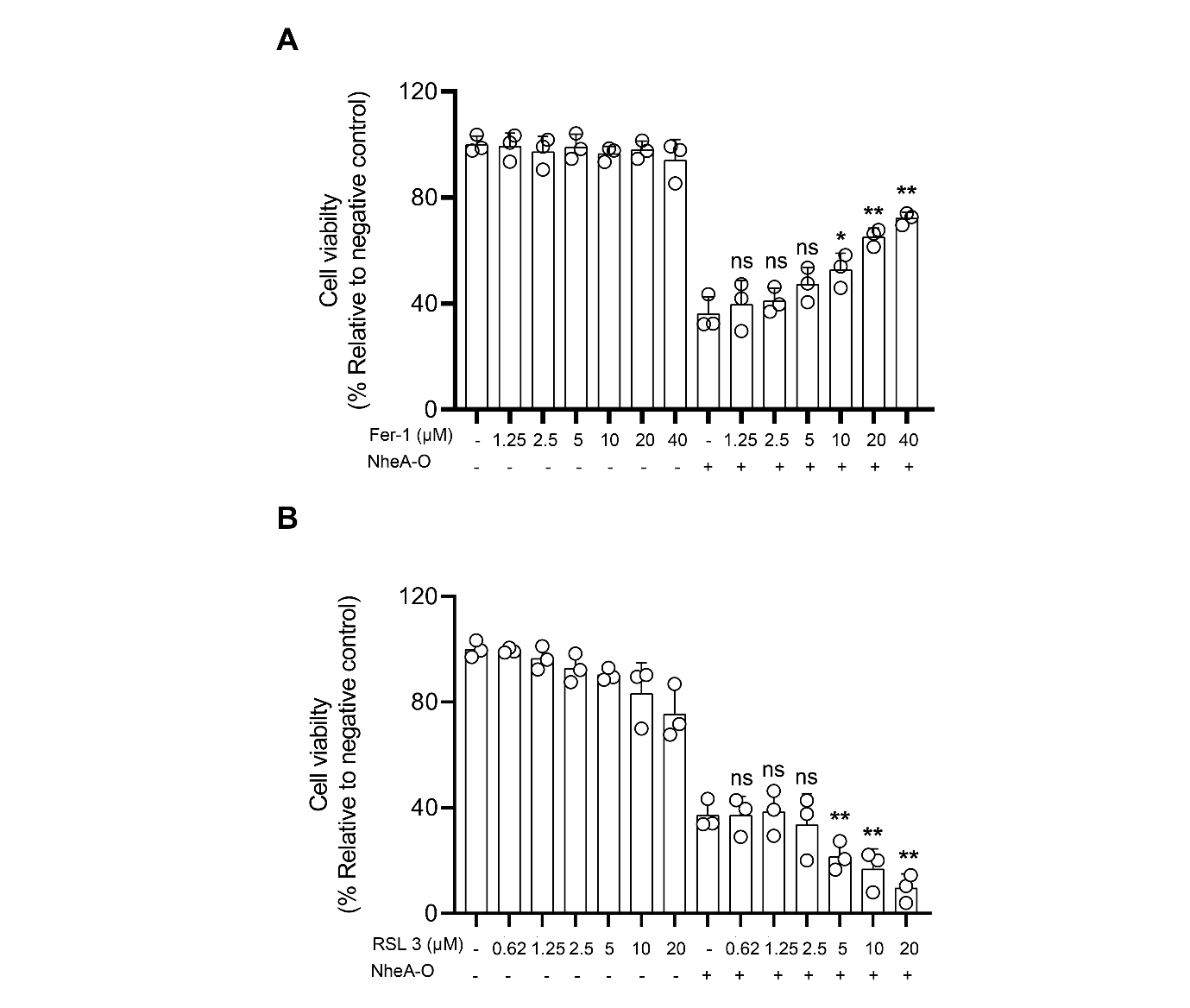


**Fig. S8: NheA-O induces ferroptosis in tumor cells. (A-B)** NheA-O mediated cell death in DLD1 colorectal cancer cells in the presence or absence of ferroptosis inhibitor (Fer-1) or activator (RSL3). Cell viability was quantified by MTS cell viability assay (n = 3; biological replicates). Bar graphs show mean ± s.d. Significance was determined from replicates using a one-way analysis of variance (ANOVA) with Dunnett’s post-test against NheA-O treated cells. ***p < 0.01, *p ≤ 0.05, ns = non-significant.*

**
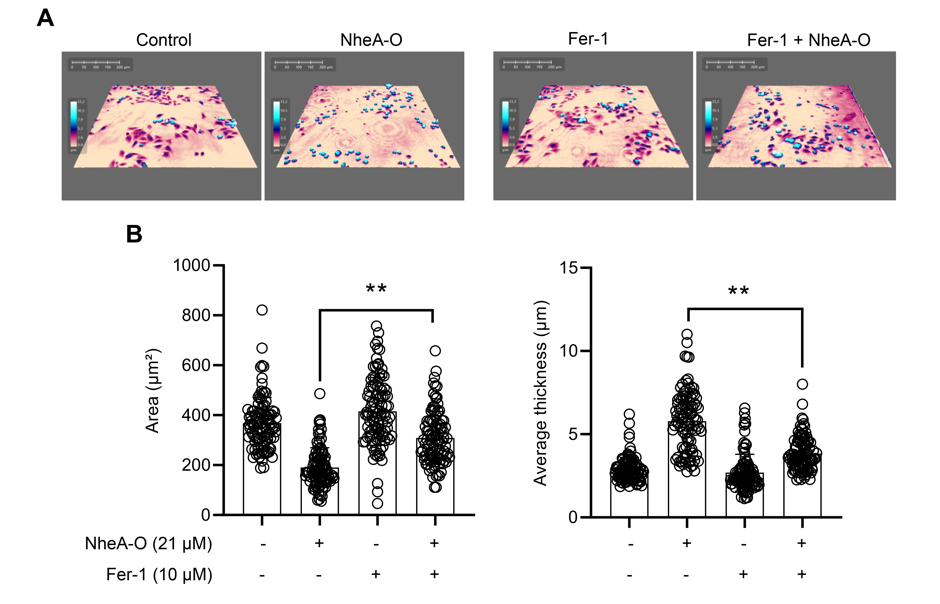
**

**Fig. S9: Ferroptosis inhibitor rescues NheA-O effect on colorectal cancer cells. (A)** Representative holographic images of DLD1 cells exposed to NheA-O in the presence or absence of Fer-1. **(B)** Histograms indicate quantification of area and thickness of images presented in panel (A). Bar graphs show data from two biological replicates (n = 50 cells). Data is represented as mean ± s.d. Significance was determined using unpaired Student’s t-test. ***p < 0.01, *p ≤ 0.05.*


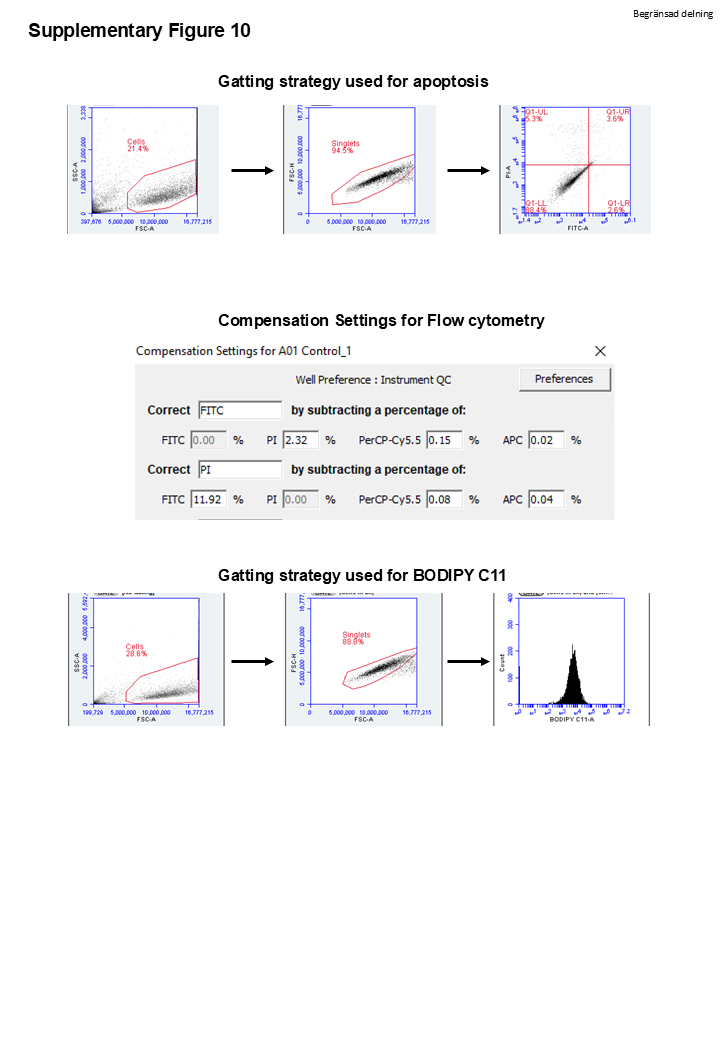


**Fig. S10: Gatting strategy used for flow cytometry**


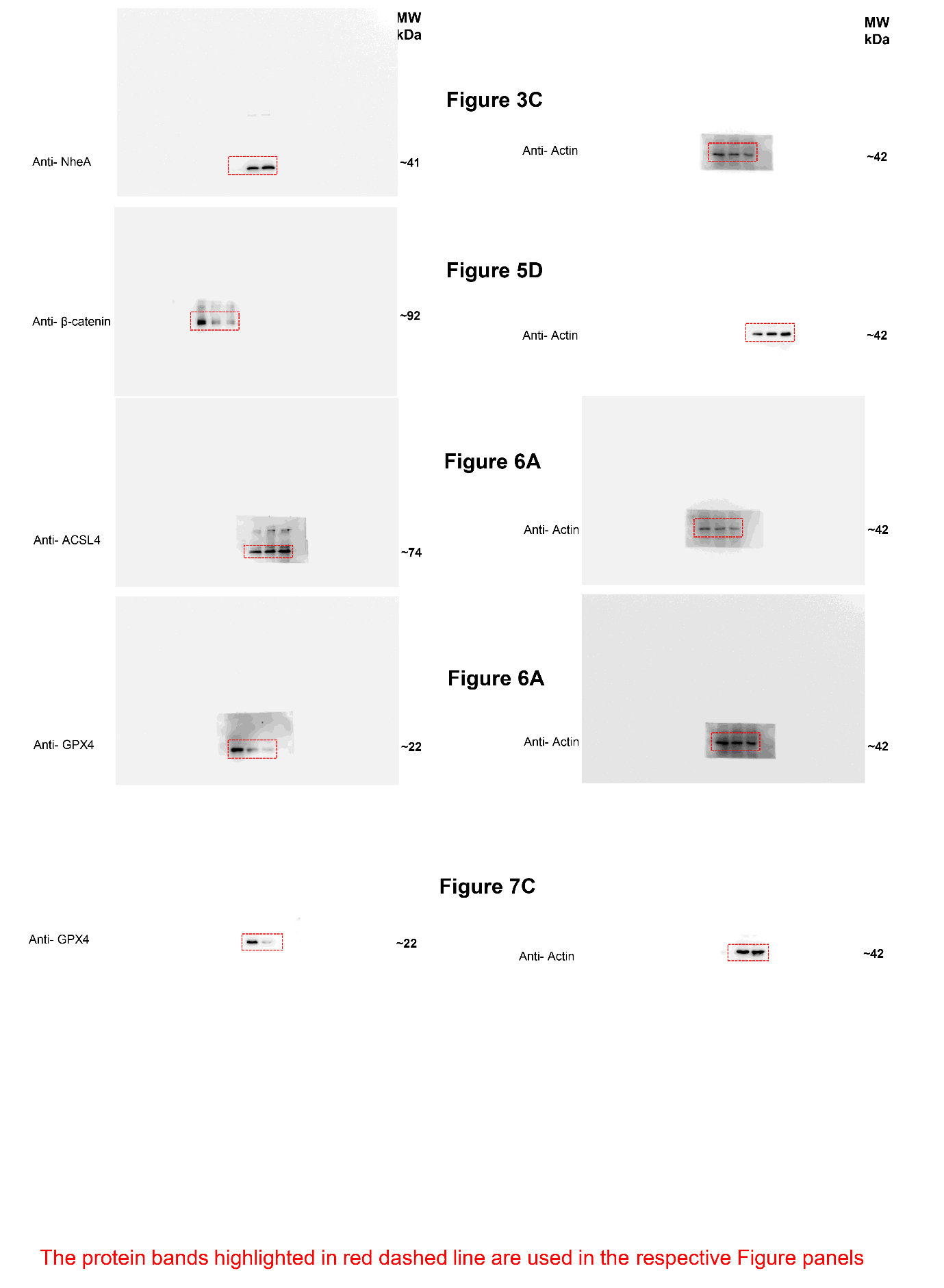


**Fig. S11: Uncropped WB images**


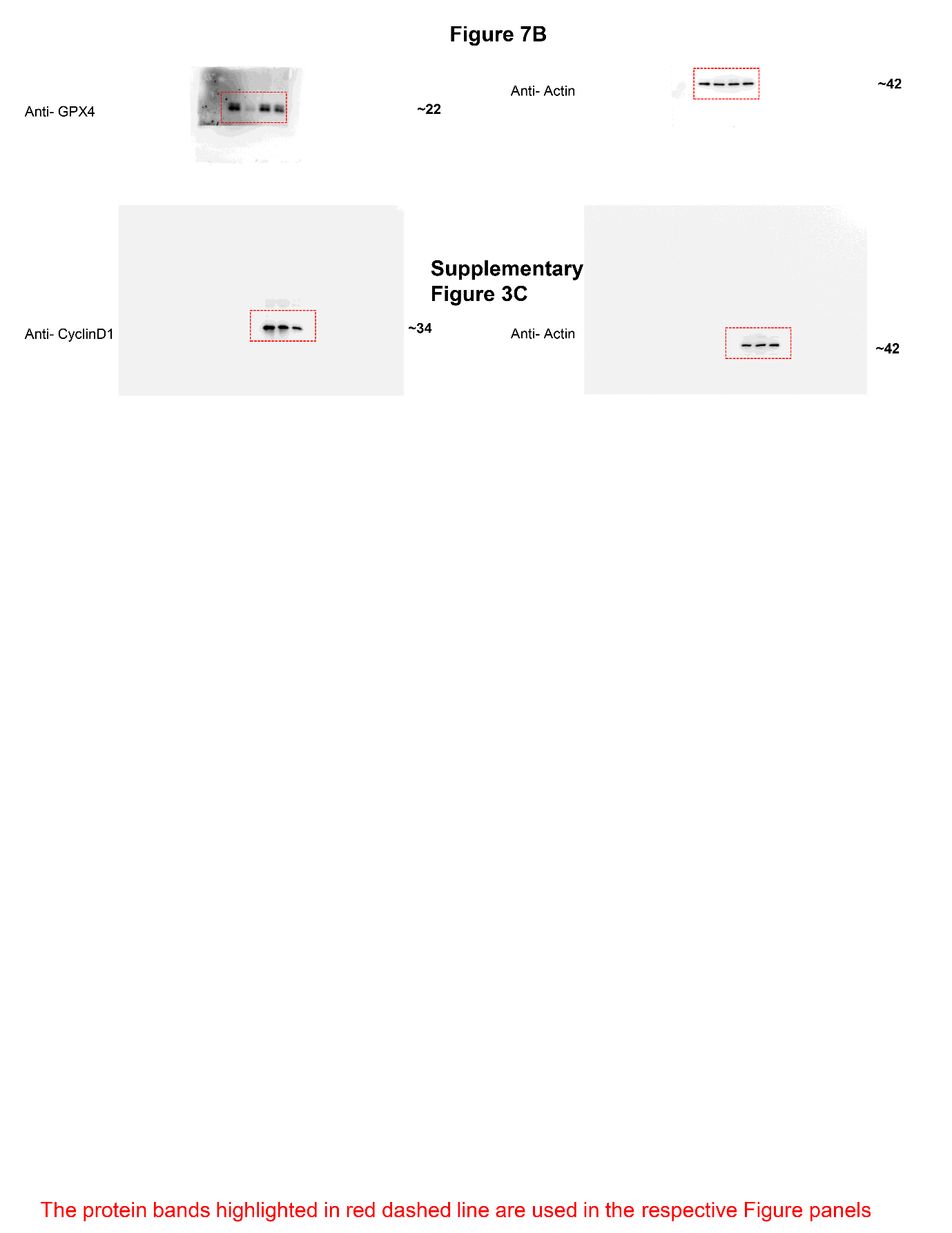


**Fig. S11: Uncropped WB images**
