# Supplementary material for: Bacterial protein-oleate complexes induce ferroptosis-like cell death in colorectal cancer cells by disrupting cell membranes and inhibiting the β-catenin-GPX4 axis
Source: Cell Death Discov. 2026 Apr 11;12:182. doi: 10.1038/s41420-026-03097-9 (PMC13076691; doi:10.1038/s41420-026-03097-9)
Supplement: Supplementary file 2 — Uncropped Western Blots [file 41420_2026_3097_MOESM2_ESM.pdf]

Supplementary Figure 11

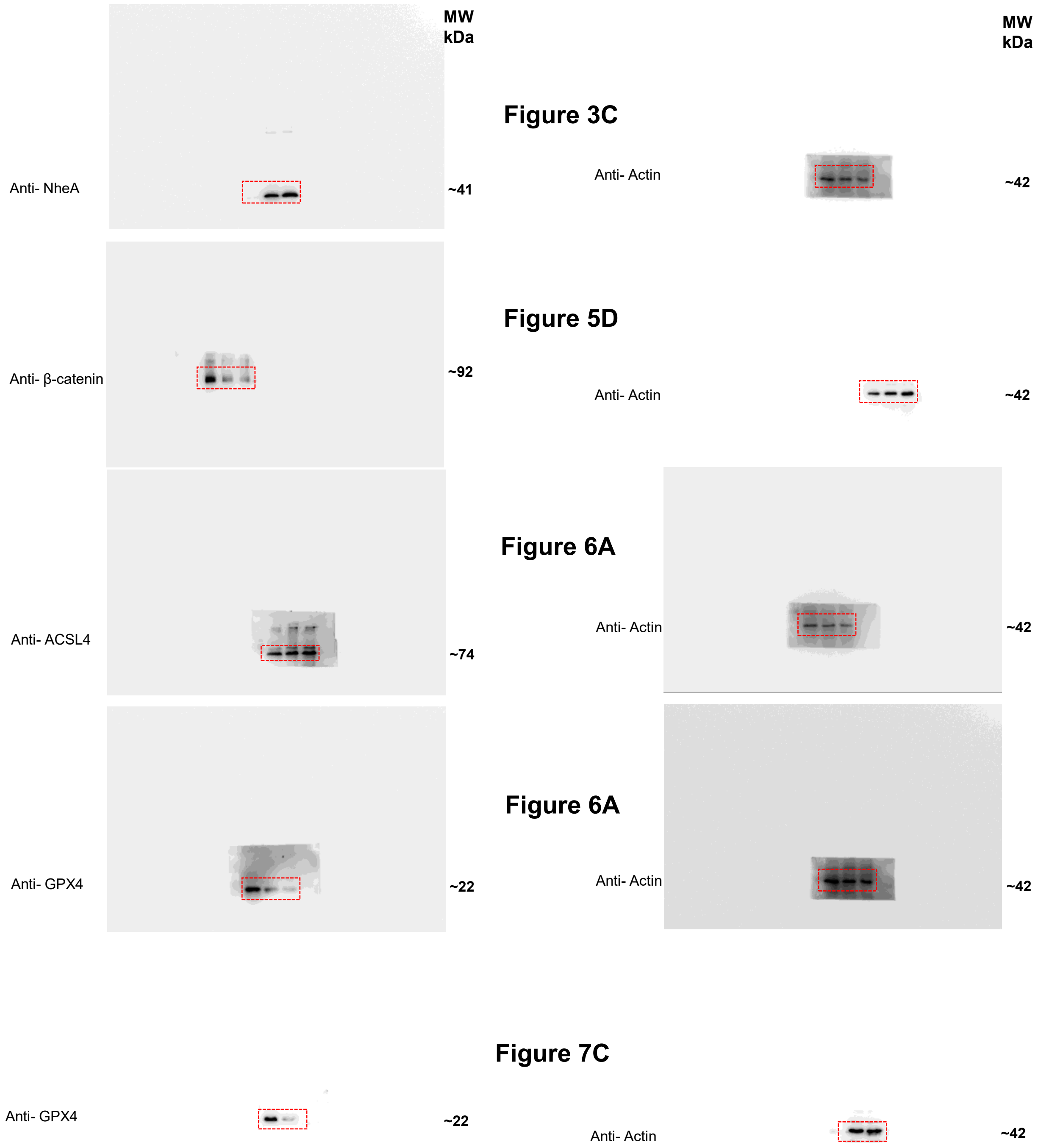

The protein bands highlighted in red dashed line are used in the respective Figure panels

Supplementary Figure 11

Figure 7B

Anti- GPX4

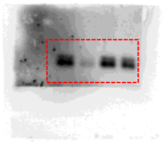

~22

Anti- Actin

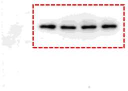

~42

Supplementary Figure 3C

Anti- CyclinD1

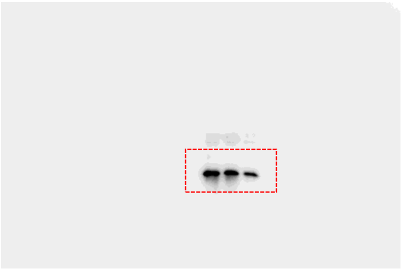

~34

Anti- Actin

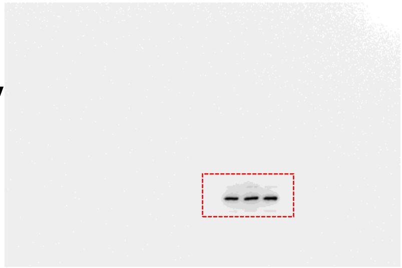

~42

The protein bands highlighted in red dashed line are used in the respective Figure panels
